# Uncovering mechanisms behind mosquito seasonality by integrating mathematical models and daily empirical population data: *Culex pipiens* in the UK

**DOI:** 10.1186/s13071-019-3321-2

**Published:** 2019-02-07

**Authors:** David A. Ewing, Bethan V. Purse, Christina A. Cobbold, Stefanie M. Schäfer, Steven M. White

**Affiliations:** 1 Centre for Ecology & Hydrology, Benson Lane, Crowmarsh Gifford, Wallingford, OX10 8BB UK; 2 Department of Mathematics and Statistics, University of Glasgow, University Place, Glasgow, G12 8QQ UK; 3 Present address: Biomathematics and Statistics Scotland, James Clerk Maxwell Building, Peter Guthrie Tate Road, The King’s Buildings, Edinburgh, EH9 3FD UK; 4 The Boyd Orr Centre for Population and Ecosystem Health, University of Glasgow, University Avenue, Glasgow, G12 8QQ UK; 5 The Wolfson Centre for Mathematical Biology, Mathematical Institute, Radcliffe Observatory Quarter, Woodstock Road, Oxford, OX2 6GG UK

**Keywords:** *Culex pipiens*, Density-dependent seasonality, Delay-differential equation, Vector modelling, Stage-structured modelling, West Nile virus

## Abstract

**Background:**

Many mosquito-borne diseases exhibit substantial seasonality, due to strong links between environmental variables and vector and pathogen life-cycles. Further, a range of density-dependent and density-independent biotic and abiotic processes affect the phenology of mosquito populations, with potentially large knock-on effects for vector dynamics and disease transmission. Whilst it is understood that density-independent and density-dependent processes affect seasonal population levels, it is not clear how these interact temporally to shape the population peaks and troughs. Due to this, the paucity of high-resolution data for validation, and the difficulty of parameterizing density-dependent processes, models of vector dynamics may poorly estimate abundances, which has knock-on effects for our ability predict vector-borne disease outbreaks.

**Results:**

We present a rich dataset describing seasonal abundance patterns of each life stage of *Culex pipiens*, a widespread vector of West Nile virus, at a field site in southern England in 2015. Abundance of immature stages was measured three times per week, whilst adult traps were run four nights each week. This dataset is integrated with an existing delay-differential equation model predicting *Cx. pipiens* seasonal abundance to improve understanding of observed seasonal abundance patterns. At our field site, the outcome of our model fitting suggests interspecific predation on mosquito larvae and temperature-dependent larval mortality combine to act as the main sources of population regulation throughout the active season, whilst competition for resources is a relatively small source of larval mortality.

**Conclusions:**

The model suggests that density-independent mortality and interspecific predation interact to shape patterns of mosquito seasonal abundance in a permanent aquatic habitat and we propose that competition for resources is likely to be important where periods of high rainfall create transient habitats. Further, we highlight the importance of challenging population abundance models with data from across all life stages of the species of interest if reliable inferences are to be drawn from these models, particularly when considering mosquito control and vector-borne disease transmission.

**Electronic supplementary material:**

The online version of this article (10.1186/s13071-019-3321-2) contains supplementary material, which is available to authorized users.

## Background

Multiple interacting ecological processes underpin seasonal dynamics of mosquitoes, which act as important disease vectors worldwide [1]. Environmental conditions affect life history parameters in many ways, including for example, temperature effects on mortality, development and biting rates [2–5], photoperiod effects on overwintering behaviour [6–8] and hydrological effects on larval site availability and egg-laying [9]. Further, density-dependent effects of competition for resources in the immature life stages, and intra- and interspecific predation have been shown to have profound effects on seasonal abundance patterns across a range of insect species [2, 10–12]. This seasonality in vector dynamics, combined with the direct effects of environmental conditions on pathogen replication and transmissibility in the host, can lead to pronounced seasonality in human cases of vector-borne disease in both tropical and temperate climates [13, 14]. This seasonality in disease occurrence is observable in most mosquito-borne diseases, including malaria, dengue, chikungunya and West Nile virus (WNV) [13–16].

In recent years environmental change has been implicated in the expansion and increased incidence of a wide range of vector-borne diseases across Europe and this trend is expected to continue in the coming decades [17]. Range expansion of the tick, *Ixodes ricinus*, to higher latitudes and altitudes and increased incidence of Lyme borreliosis across Europe have been partially attributed to warm winters and elevated summer temperatures [17, 18]. Further, the spread of *Aedes albopictus* across Europe has been linked to trade, travel, warm seasonal and annual temperatures, and ample rainfall [19, 20] and has been linked to outbreaks of dengue in France and Croatia in 2010 [21, 22] and five outbreaks of chikungunya in France and Italy between 2007 and 2017 [23–27]. However, we can only fully understand and make predictions regarding mechanisms linking these environmental changes to increases in disease incidence and geographical distribution if we develop models that incorporate both biotic and abiotic drivers of vector abundance and disease transmission [28]. This underlying seasonality in vector abundance has been shown to be a key driver of the seasonality observed in vector-borne diseases [13, 29, 30].

Mosquito-borne disease is a massive global health burden, with almost 350 million estimated cases of mosquito-borne disease across the globe in 2017 [31]. Many mosquito-borne diseases exhibit substantial seasonality, which can be at least partly attributed to seasonality in the vector population [13]. The phenology of mosquito populations is complex and driven by a range of factors that need to be understood to determine how mosquito seasonality might drive disease transmission. Abiotic factors such as temperature, that have density-independent effects on population dynamics, may have opposing impacts on particular demographic rates. For example, high temperatures can have a negative effect on adult mosquito survival [32, 33] but a positive effect on immature development [5, 33]. Biological processes such as intraspecific competition can have density-dependent effects on vector population dynamics and abundance, with limited access to food or other resources being shown to prolong development times and decrease survival [2, 34]. Interspecific interactions, such as predator-prey interactions or competition between mosquito species can also have density-dependent effects on population dynamics and phenology, potentially acting to suppress population sizes even when environmental conditions are favourable [35, 36]. The importance of these processes may show geographic variation dependent on local environmental conditions including topography, climate, hydrology, habitat and species compositions of predators and competitors.

Recently, large-scale studies using statistical models to investigate the relative contributions of density-dependent and density-independent processes to vector population dynamics have become more common, with a recent study by Chaves et al. [37] showing that population dynamics of *Aedes aegypti*, exhibited both substantial density-dependent regulation and sensitivity to temperature and rainfall. Numerous recent studies of *Culex pipiens* in northern Italy have found density-dependence to be an important determinant of abundance patters. Jian et al. [38] monitored adults at more than 20 sites over two years and found significant density-dependence at the time scale of mean larval development using a Gompertz-logistic based model, whilst Mulatti et al. [39] found that density-dependence contributed more strongly to vector population growth rates than any single environmental factor across three years of adult captures using a similar model structure. However, whilst these statistical models are useful in identifying the presence and timescale of density-dependence in adult capture data, it is not straightforward to extend these findings to accurately incorporate patterns of intra- and interspecific density-dependence throughout the year in mechanistic models.

Failing to quantify these density-dependent processes in mechanistic vector population models will hamper our ability to predict vector phenology. However, a sound understanding of vector phenology is necessary to accurately predict disease transmission seasons, transmission intensity and disease persistence between years, as well as the potential outcomes of vector control strategies [40–42]. Understanding the relative importance of density-dependent and density-independent factors on mosquito abundance is crucial because different life stages inhabit different ecological niches and therefore may be subject to different control measures [42–44]. For example, White et al. [40] use a stage-structured mathematical model for *Ae. aegypti* to show that whilst high frequency releases of transgenic males will reduce adult mosquito abundance in many cases, the use of sterile insect techniques (SIT) alone can lead to population increases in some instances. These increases stem from the dynamics in the aquatic larval stage, which is not targeted by SIT, and are due to the reduction in natural larval density-dependent mortality caused by reduced egg-laying. Consequently, both the ecological niche targeted and the seasonal timing of releases proved to be highly influential. White et al. [41] showed that density-dependent regulation of anopheline mosquito larvae can ensure that persistence of mosquito populations is robust against control measures targeting the individual larval, pupal and adult life stages and that a suite of interventions that target different stages of the life-cycle is necessary to ensure maximum reductions in mosquito density.

The treatment of vector seasonality in mosquito-borne disease models varies considerably, from assumptions of constant mosquito populations, to detailed seasonal patterns and specific behaviour patterns [15]. In poorly studied and data-sparse systems, modellers will commonly ignore seasonality in favour of studying population dynamics at equilibrium using classical *R*_0_ methodologies [15, 45]. However, increasing awareness of climate sensitivities and impacts of environmental change on insect vectors and vector-borne disease has led to an increased number of models being developed such that vector dynamics are climate-forced, using functions that relate population growth parameters to environmental variables [15, 46–48] in an attempt to capture the seasonality in patterns of mosquito-borne disease. Due to the complexities associated with modelling multiple abiotic and biotic population drivers, many model systems neglect density-dependence, with a recent review paper finding that only 7.5% of 373 disease models published between 1970–2010 that incorporated mosquito dynamics explicitly accounted for density-dependence in the aquatic stage [45]. Such processes are challenging to model, particularly as surveillance usually focuses on the monitoring of only the adult stage during the peak transmission season [38, 39, 49] because it is adult females which transmit disease, adults are comparatively easily trapped and surveillance resources are often constrained [50].

Vector population models are often built using life history data derived from laboratory studies and can be validated quite crudely, for example by qualitatively comparing the timing of abundance peaks or first appearance and disappearance of adults in weekly or monthly trap catches [48, 51, 52]. This reliance on adult data means that it is unclear whether models correctly capture the demographic processes of the immature stages. For example, the study by White et al. [41] investigating the effects of density-dependent larval mortality on control of anopheline mosquitoes, was conducted using a theoretical model which was parameterised using only adult capture data from eight villages in Nigeria, meaning the effects of control measures targeting immature life stages were not assessed by direct comparison with empirical evidence relating to the immature stages. Further, the aforementioned large-scale statistical models [37–39] investigate the effects of density-dependence on the adult population using adult capture data without empirical data on the density-dependent aquatic stages themselves. By improving understanding of the factors which drive seasonal abundance and transmission patterns we aim to produce more robust predictions of disease seasonality allowing better assessment of the likely effectiveness of proposed control measures.

In this study we focus on *Cx. pipiens* which has been widely implicated as a major vector of WNV [1, 53–55] and is common across much of the UK, prompting concerns that disease outbreaks may occur if the disease were to be introduced [56]. WNV is the most significant cause of mosquito-borne disease in temperate regions including Europe and North America [57–59] and transmission of the virus is highly seasonal, with human cases typically peaking in late summer and early autumn [60]. This seasonality in temperate climates is believed to stem from the fact that low temperatures over winter months create conditions unsuitable for development and survival of immature mosquitoes, causing a cessation of breeding activity as adults enter a diapausing state [61], whilst low temperatures cause conditions unsuitable for replication of the virus within exposed vectors [4]. Hence, accurate predictions of WNV occurrence and dynamics will depend on our ability to accurately predict *Cx. pipiens* seasonal abundance, which we aim to address here.

We present a high temporal resolution dataset of seasonal abundance of all life stages of *Cx. pipiens*. This dataset is thoroughly explored using a delay differential equation (DDE) model developed from that published by Ewing et al. [62]. This model allows the duration of, and survival through, each mosquito life stage to vary in response to temperature, whilst diapause initiation and termination are primarily governed by photoperiod. Ewing et al. [62] predict that *Cx. pipiens* abundance will increase in the UK under current climate projections and that the timing of warm periods may be particularly influential in shaping abundance patterns. However, the model used was not validated with data and omitted larval competition for resources, which may be an important driver of abundance patterns. We further develop the model by including larval competition for resources and by allowing the strength of predation on larvae to vary through the season. This model is then used to explore and understand patterns of abundance seen in the field data. By using the field data in conjunction with the population model we investigate the relative contributions of interspecific predation, competition for resources and abiotic factors to seasonal patterns of immature survival and mosquito abundance. This allows us to investigate the importance of individual density-dependent and -independent processes throughout the year, improving understanding of how processes interact to shape phenology across the duration of a season. We also assess the ability of the model to capture abundance patterns across all four life stages to better understand the appropriateness of the common approach of validating models against only adult data.

## Methods

### Field data collection

**Fig. 1:**
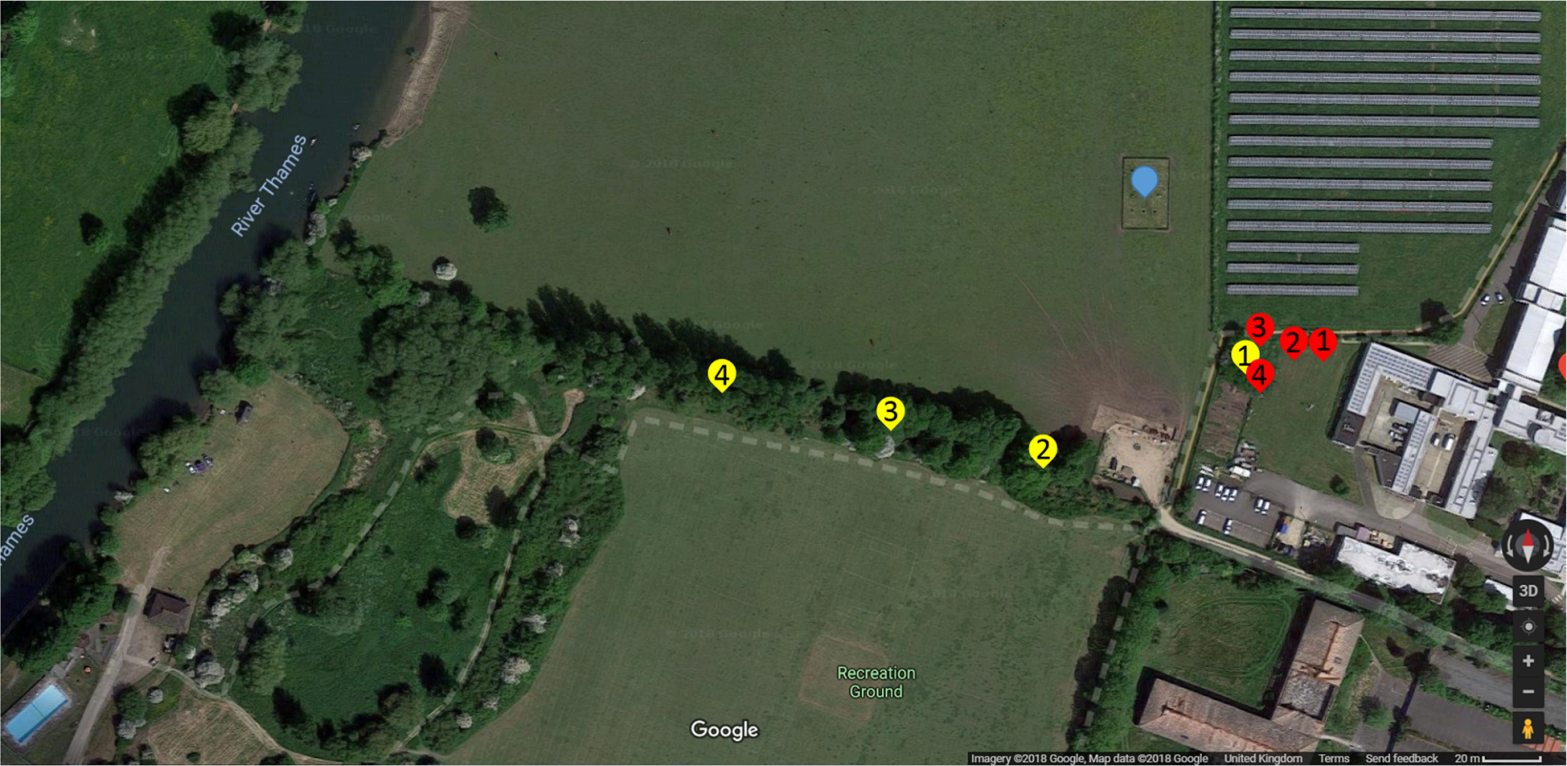
Field site. Red markers show the locations of water butts 1–4. Yellow markers show the locations of adult traps 1–4. The blue marker shows the location of the meteorological site. Adult traps 2, 3 and 4 are a distances of approximately 80 m, 140 m and 200 m from the water butts. Imagery ©2018 Google, Map data ©2018 Google

Intensive sampling of adult and immature mosquitoes was carried out from the 2nd of March until the 5th of October, 2015. This start date was one month earlier than start dates of prior UK studies of adult populations [63–65] and was chosen to ensure that the start of the *Cx. pipiens* season would be captured. Collections were stopped at the beginning of October, once no eggs or adults had been observed for five consecutive sampling occasions. The field study was carried out on the grounds at the Centre for Ecology & Hydrology (CEH) site in Wallingford (51°36'9.0144"N, 1°6'45.7344"W) (Fig. 1).

Immature *Cx. pipiens* were monitored using four 450 l circular water butts placed at the locations shown in Fig. 1. Although within 20 m of each other, the four butts varied in biotic and abiotic conditions. Butts number 1 and 2 were placed in exposed locations with some shelter provided by a hedge/hedgerow on the northern side but all other sides open and no overhanging vegetation. These butts received direct sunlight throughout most of the day. Butt number 3 was more sheltered with no overhanging vegetation but cover on the north and west sides and direct sunlight until late afternoon or early evening depending on the time of year. Butt number 4 was sheltered under a tree and only received direct sunlight in the morning. In January prior to the start of sampling, each butt was filled with 450 l clean water, which was then infused with hay by suspending 2 kg of hay in a net bag. The hay infusion was used because adult female *Cx. pipiens* are known to favour breeding sites with a high organic matter content and hay infusions have been shown to be effective attractants [66–68]. The bags of hay were left in the butts until the end of March when the water had become visibly organic matter-rich and there was discernible algal growth around the edges of the butts. The hay bags were then removed for ease of sampling to avoid the larvae hiding in the hay bags. The organic content in the water butts were not controlled throughout the season to allow mosquito seasonal abundance to be observed under naturally varying conditions. A HOBO temperature logger, floating on the surface of the water, was set to record surface water temperature at hourly intervals in each butt, as *Cx. pipiens* spend most time at the surface [69].

The number of egg rafts in each butt was counted at 10:00 h on Mondays, Wednesdays and Fridays, allowing abundance changes to be tracked at a high temporal resolution (note that in three weeks only two counts were made due to logistical issues). On each sampling occasion, after the egg rafts had been counted, a 500 ml dip was taken from each of the north, south, east and west edges of each water butt using the standard dipping procedure (described by Fillinger et al. [43]). No dips were taken from the middle of the butt as both larvae and pupae were observed to congregate at the edges. The dips were transferred into one or more white plastic trays and all aquatic life stages of the mosquitoes were counted and recorded. Digital photographs were taken of the contents of each tray and the samples were then counted manually on the computer to avoid counting error due to the movement of live larvae or pupae in the field. The validity of counting from photos was checked by comparing direct counts from the tray with counts from photos on the first two days of sampling and all counts from the photographs were found to be consistent with direct counts from the field. All specimens were returned to the water butts after photographing to prevent removal effects from one catch to the next. Twenty fourth-instar larvae were taken from each water butt (when abundances were high enough that the number removed was a small proportion of the total population) monthly for morphological identification to species level [70]. Those fourth-instar larvae taken for identification were examined by microscopy in the laboratory using the identification keys in Becker et al. [70]. Larvae were killed prior to examination by submersion in boiling water.

To sample the adult population four CDC Miniature Downdraft Blacklight (UV) Traps (Model 912, John W. Hock company, USA) were run from the 14th of April until the 2nd of October, by which 5 consecutive zero collections had been recorded, in the yellow locations indicated in Fig. 1. The traps were baited with dry ice to attract female adult mosquitoes and were run 4 times a week overnight from Monday to Thursday throughout the year (though seven nights were missed due to logistical issues). Adult trapping started later than immature trapping due to logistical issues with the supply of dry ice. Trap 1 was hung amongst trees adjacent to the water butts and traps 2 to 4 were hung in the tree line at the side of an adjacent field, in which cattle were grazed, at distances of approximately 80 m, 140 m and 200 m from the water butts (Fig. 1). The traps were run from 17:00 h each day until 9:00 h the following morning, as *Cx. pipiens* biting peaks just after sunset and during sunrise [71]. Adults were placed in the freezer immediately after collection and left for at least one hour before identification. All mosquitoes caught were identified to species level by microscopy in the laboratory using morphological identification keys [70] and the number of females of each species was recorded. Males were not recorded as they are nectar-feeders, do not contribute directly to disease spread, and are underrepresented in catches of light traps that use CO_2_ to simulate host cues. Minimum and maximum daily ambient air temperature, cumulative daily rainfall, daily sunlight hours and mean daily wind speed were recorded throughout the sampling period at a CEH weather station in the adjacent field.

### Modelling to understand the role of developmental processes and mortality rates in explaining observed abundance patterns

**Fig. 2:**
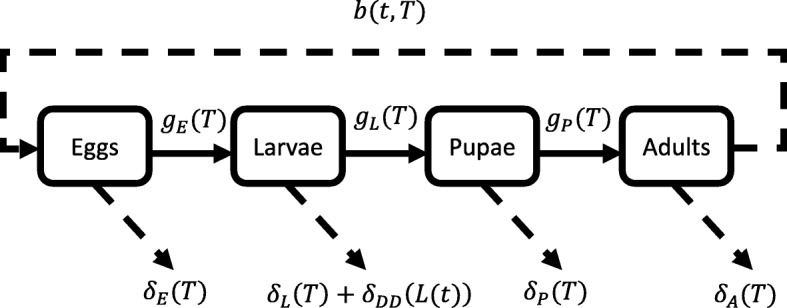
A flowchart of the DDE model, where *g*_*i*_(*T*) represents the development (or growth) rate and *δ*_*i*_(*T*) denotes the temperature-dependent mortality rate of individuals in stage *i* (*i* = *E*, *L*, *P*) at temperature, *T*. The additional mortality term experienced by larvae, *δ*_*DD*_(*L*(*t*)), denotes the rate of mortality due to density-dependent processes, which may be due to either predation or competition for resources. In the model presented by Ewing et al. [62] density-dependence occurs through a Holling type II predation function (*δ*_*DD*_(*L*(*t*)) = *δ*_*π*_(*L*(*t*))) but other density-dependent processes could be modelled, so we write this as a general density-dependent term, *δ*_*DD*_(*L*(*t*)) = *δ*_*π*_(*L*(*t*)) + *δ*_*LC*_(*L*(*t*)), composed of a predation term and a larval competition term. The egg-laying rate at which adults lay new offspring is given by *b*(*t*, *T*), such that the egg-laying rate depends on the duration of the temperature-dependent gonotrophic cycle and whether mosquitoes are active or diapausing, which varies with time, *t*

The model used here is based upon the stage-structured variable-delay-differential equation (DDE) model presented in Ewing et al. [62] and was used to predict seasonal abundance of *Cx. pipiens* under temperature and photoperiod conditions measured at the field site. This generic variable DDE framework was originally developed by Nisbet & Gurney [72] and was applied to a general insect population where food availability affected the duration of the immature stage. The model was extended by Ewing et al. [62] to incorporate the differential effects of temperature on the duration and mortality of each mosquito life stage, such that the total duration of, and survival through, each life stage varies in response to changes in temperature. The population was also assumed to undergo density-dependent regulation through interspecific predation on larvae. Further, the adult dynamics are affected by both temperature and photoperiod through their respective effects on egg-laying rates and diapause behaviour. Here, we further extend the model, shown in Fig. 2, to include the effect of larval competition on mortality and to account for potential seasonality in the ratio of predators to prey. Consequently, both density-dependent and density-independent mortality will act on the model simultaneously, with the relative strengths of these processes being dependent on the temperature, time of year and population size. Any modifications to the Ewing et al. [62] model are discussed in detail in Additional file 1: Text S1-4, which gives a description of all functional forms, parameter values, initial conditions, and history and inoculation values used in conducting model simulations. The functional forms presented by Ewing et al. [62] were fitted to data regarding *Cx. pipiens* vital rates from the existing literature. Temperature-dependent mortality, development and egg-laying rates were derived from laboratory studies, whilst photoperiod thresholds governing diapause initiation and termination were determined from field studies monitoring adult populations in overwintering shelters. In all model simulations the air temperature measured at the CEH weather station and the hourly water temperatures measured in butt 4 were used, as butt 4 maintained a high abundance of mosquitoes throughout the season. We solve the system of DDEs in Fortran 90 using the DDE solver (DDE_SOLVER) written by [73]. The code for the model described here can be found at [74]. The most significant alterations to the Ewing et al. [62] model are the inclusions of larval competition and seasonally forced predation on larvae as well as changes to the timing of entry and exit to diapause and a post-diapause adult female mortality (see results for rationale).

### Larval competition

Competition has been shown to affect mortality of larvae of a range of mosquito species in numerous laboratory and field studies [2, 34, 75, 76], with several functional forms, including linear, exponential, log-linear and quadratic forms, suggested to capture this process [77, 78]. Consequently, a density-dependent larval competition function was added to the Ewing et al. [62] model. This function was fitted to data published by Madder et al. [2] on the effects of competition on larval survival in *Cx. pipiens* and the exponential function was found to give the best fit (Additional file 1: Text S2.3 and Figure S1), consistent with the findings of Beck-Johnson et al. [79] for *Anopheles* mosquitoes. Accepting the exponential functional form, we also fitted the competition parameters directly using the field data and compared experimental estimates of competition with field data estimates. We make the simplifying assumption that larval competition only affects mortality rates. Previous studies have shown that development times, body size of adults, female fecundity, and adult longevity can all be impacted by competition in the larval stage [80]. Consequently, incorporating these non-lethal effects of competition may increase our understanding of how density-dependent effects combine to shape abundance patterns. However, existing studies examine these effects in isolation, making the simultaneous quantification of effects of competition on, for example, mortality and development rates difficult. As this is the first study using this DDE modelling approach to incorporate the effects of predation, competition and density-independent effects on vector seasonal abundance, we decided to focus solely on the effects of competition on larval mortality, which has been observed to be the strongest competition outcome [10, 81].

Our aim was to investigate seasonal fluctuations in the mosquito population under natural variation in a field habitat and to understand if these population fluctuations could be captured using relationships from existing studies. Therefore, we increased the organic resources in the butts at the start of the season to provide good larval habitat but did not attempt to control the organic matter content further afterwards.

### Seasonal patterns in predation

Interspecific predation of *Cx. pipiens* larvae has commonly been found to follow a Holling type II pattern in laboratory studies [82–84], as used by Ewing et al. [62]. However, the ratio of predators to prey, which could not be estimated from the field data, is likely to be variable throughout a year dependent on the seasonality of the wide range of species which may predate on *Cx. pipiens* larvae [68, 85]. During the fieldwork a variety of known mosquito larval predators including backswimmers, water boatmen and damselfly larvae were observed. Consequently, the potential effects of seasonality in the predator to prey ratio were investigated by simulation using a sinusoidal wave whereby the ratio of predators to prey was allowed to increase throughout spring before peaking in summer. This sinusoidal wave is considered to represent a group of generalist predators which feed upon *Cx. pipiens* larvae. The functional form of the seasonal function is presented in Additional file 1: Text S2.4 and shown in Figure S2. Parameters driving the seasonality of the predator population could not be derived from the literature and were therefore fitted to the field data.

### How the DDE model was used to investigate the role of DI and DD mortality in producing field observations

Predictions from the DDE model were compared with the field data to assess model performance and investigate the relative roles of density-dependent and density-independent mortality to mosquito abundance patterns. The three parameters governing seasonality in predator numbers could not be estimated from lab or pre-existing field data, so these were fitted to the field data using approximate Bayesian computation (ABC) rejection sampling [86]. These parameters were the maximum number of predators per larva during the season, *r*_*max*_, the time at which this peak predator density was reached, *υ*, and the parameter which scaled the duration of this relatively high predator abundance, χ. Uniform priors were placed on each parameter: *r*_*max*_~*U*(0,0.025) where the upper bound of 0.025 was chosen to coincide with sufficiently high predator numbers that the population regularly crashed, *υ*~*U*(0, 60) (days) corresponding to a predator peak during July or August consistent with qualitative observations from the field and *χ*~*U*(0, 10) covering a large range of potential predation function widths. A further ABC fitting procedure was also carried out where the two parameters determining the shape of the competition function, *c*_0_ and *c*_1_, which were taken from the literature [2], were included alongside the predation parameters, with priors of *c*_0_~*U*(0,0.01) and *c*_1_~*U*(0,0.01), to better understand the ability of different combinations of the predation and competition to capture the field data. This range of priors includes the special cases of no competition (*c*_0_ = 0), constant predation (χ = 0), and no predation (*r*_*max*_= 0). ABC was used in this instance as it allows us to simulate posterior distributions for our parameters whilst bypassing evaluation of the likelihood function which would be very computationally costly, particularly under fluctuating environmental conditions. Having simulated model results using parameters drawn from the given priors, the simulated and observed data were compared using a procedure referred to as the “egg-to-pupae procedure”, which allows the roles of density-independent and density-dependent mortality in producing field observations to be examined directly.

The egg-to-pupae procedure involves simulating the DDE model using the initial conditions and history values given in Additional file 1: Text S4 and Table S1 to obtain time series of the model-predicted immature stage durations, ${\widehat{\tau}}_i(t)$, and survivals, ${\widehat{S}}_i(t)$, where *i* = *E*, *L*, *P*, given the environmental conditions at the field site and the simulated mosquito densities. We then extrapolate forward in time from the egg abundance observed in the field, *O*_*E*_(*t*), using the simulated stage duration and survival values to estimate pupal abundance, $\widehat{P}(t)$, for comparison with the field data, *via*

1 $$ {\displaystyle \begin{array}{l}\widehat{P}(t)={O}_E\left(t-\frac{1}{2}{\widehat{\tau}}_E\left(t-\frac{1}{2}{\widehat{\tau}}_P(t)-{\widehat{\tau}}_L\left(t-\frac{1}{2}{\widehat{\tau}}_P(t)\right)\right)-{\widehat{\tau}}_L\left(t-\frac{1}{2}{\widehat{\tau}}_P(t)\right)-\frac{1}{2}{\widehat{\tau}}_P(t)\right)\sqrt{{\widehat{S}}_E(t)}{\widehat{S}}_L(t)\sqrt{{\widehat{S}}_P(t)}\\ {}\\ {}\end{array}} $$

Equation 1 uses half the egg stage duration because it was not possible to identify the age of each egg raft, so we averaged across the possible ages by assuming eggs had completed half their development. Similarly, the age of observed pupae was unknown, so we again averaged across possible ages by using half of the stage duration. The egg and pupal stages were assumed to be affected only by density-independent mortality, meaning the survival through half of these stages was the square root of the survival through the whole stage. This simplifying assumption was made because these stages do not consume food and so do not compete for resources [68]. They may suffer some density-dependent predation (e.g. there is evidence for example that responses of *Cx. pipiens* pupae to aerial predators are reduced when they are in a larger group of conspecific pupae) [87] but given the scant empirical data on predation for these stages and the fact that they are short and highly temperature dependent in duration, we focused on the impact of density-dependent predation in the larval stages in this model.

An alternative approach to estimate $\widehat{P}(t)$ is to use the observed egg abundance as the initial condition for the DDE model and simulate forward in time. However, this is not possible due to the repeated measurements of egg abundance throughout the year meaning the model would need to be re-initialised with updated initial conditions and historical values at every egg observation through the year. The egg-to-pupae process described by Equation 1 allows us to make direct comparisons between the simulated pupal abundance and the corresponding field observations at all times throughout the year more directly than is possible by simply comparing the simulated abundances from the DDE model to the field observations. Any errors introduced by uncertainty and variability in model processes, such as egg-laying, are less influential under the egg-to-pupae procedure than they would be if comparing the full DDE model output as they only affect the pupal estimate through the simulated larval density. The contributing parts of Equation 1 were obtained as follows:

(i) Predictions of immature survival were obtained by solving Equation A3 for ${\widehat{S}}_i(t)$ under the environmental conditions measured at the field site and the initial conditions specified in Additional file 1: Text S4. Simulations were run for two years with the initial year discarded to remove effects of the initial conditions, as steady cycles were observed from year two onwards when the model was run over multiple years.

(ii) Predictions of larval stage duration were obtained by solving Equation A4 for ${\widehat{\tau}}_i(t)$ under the same environmental and initial conditions. The resulting outputs of steps i-ii are time series giving immature survival and stage durations for each immature life stage at all times through the year. The second year of results from a two year period were used as in step i.

(iii) These time series of stage duration and survival, which come entirely from simulation, are combined with observed egg abundances from the field using Equation 1 to estimate pupal abundance.

(iv) The observed and predicted pupal abundances were scaled by their maximum value, due to the likely but as yet unquantified difference between egg and pupal detectability and the variability in egg raft sizes, and then smoothed by taking a 7-day moving average.

(v) Predicted pupal abundances, $\widehat{P}(t)$, were then compared against the field observations of pupal abundance, *O*_*P*_(*t*), by calculating the Euclidean distance between the two times series having applied dynamic time warping (DTW) [88] using the *dtw* package in R [89]. DTW was used when comparing time series because this allows the potential differences in timing between series to be accounted for, whereas techniques which directly compare values at given time points, such as route mean squared error, were observed to select parameters that fail to capture observed peaks over models that predict these peaks at the wrong time. DTW allowed us to choose predation parameters that correctly predicted the sequence and magnitude of peaks even if the stage durations were over- or under-estimated.

Due to the required scaling of observed and predicted abundances, it was not possible to assess the exact magnitude of survival estimates; however, relative patterns of survival across the season could be assessed.

The ABC process was carried out to determine values for the three predation parameters, *r*_*max*_, *υ* and χ. 15,000 simulations were conducted (after which point further simulations did not qualitatively affect the posterior distributions) and the 1% of parameter combinations with the lowest Euclidean distance between observed and simulated pupal abundances after applying DTW were taken as the posterior distribution of each of the three parameters. The warping applied to map the model predictions using the fitted parameter values to the field observations is discussed in Additional file 1: Text S5 and shown in Figure S3. Parameter estimates were then taken as the medians of these posterior distributions, consistent with the use of a quadratic loss function. This process was then repeated for a further 60,000 simulations including the three predation parameters and the two competition shape parameters *c*_0_ and *c*_1_, and the 0.1% of parameter combinations with the lowest Euclidean distance formed the posterior distribution. A smaller percentage of parameter combinations was kept in this case because the increased number of parameters to fit led to a greater proportion of poor fits to the field data, so a combination of a larger number of simulations and a reduced acceptance threshold was necessary to obtain meaningful posteriors.

We also directly compared the full DDE model to the observed abundance data by comparing model predictions of abundance to field observations for each life stage, where both simulated and observed time series for each life stage were smoothed by taking a centered 7-day simple moving average and scaled by their respective maximum values. All parameter values used for the simulations are given in Additional file 1: Table S1.

## Results

### Species composition of immature and adult mosquito communities

**Fig. 3:**
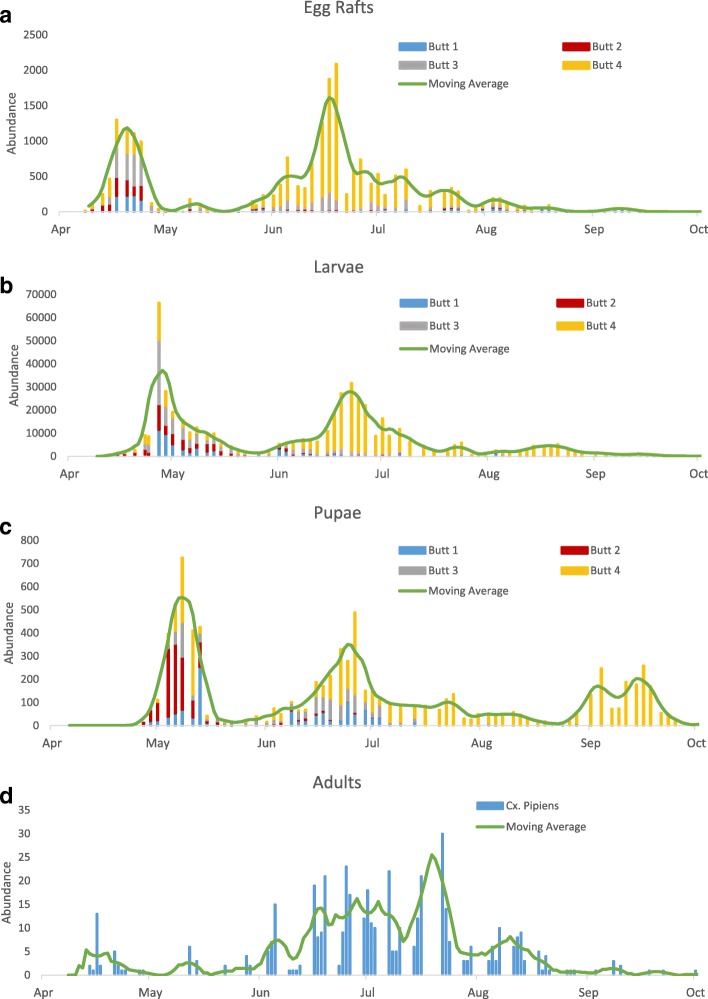
Seasonal abundance of each life stage of *Cx. pipiens*. **a** Egg rafts (first recorded on 6th of April and last recorded on 21st of September). **b** Larvae (first recorded on 15th of April and last recorded on 5th of October). **c** Pupae (first recorded on 24th of April and last recorded on 5th of October). **d** Adult female *Cx. pipiens* catch numbers collected by trap 1. In each immature plot the green line shows a centered seven day moving average of the total catch size across all butts and in the adult plot it shows a seven-day moving average of the catch from trap 1. For all immature stages there were only two observations per week on the first two weeks in July, on the 6th, 9th, 13th and 16th. Adult collections could not be taken on the following dates: 30th April, 4th and 5th May, 16th, 20th and 30th July due to problems with the supply of dry ice

In the initial immature population peak in April, the population was spread quite evenly between the four butts (Fig. 3a-c). After this initial peak in abundance, the egg population becomes concentrated in butt 4. Consequently, model simulations are compared to data from butt 4 since this contained a high proportion (76%) of all eggs laid after the initial population peak. Butt 4 had cooler surface temperatures (2 °C cooler than butts 1 and 2) due to surface shading and had higher organic matter content, which *Cx. pipiens* are known to favour [68], indicated by a higher abundance of rat-tailed maggots and a lower abundance of mayfly larvae than butts 1–3. Over the course of the sampling, morphological identification was performed on 300 fourth-instar larvae from across the 4 water butts, all of which were *Cx. pipiens*. Since all final instar larvae identified were *Cx. pipiens*, it was assumed that earlier instars were also *Cx. pipiens*. Given that *Culiseta annulata* and *Cx. pipiens* favour similar larval habitats [90] it is likely that there were also some *C. annulata* larvae present in the butts. However, as none appeared in the fourth-instar larval samples and they accounted for only 5.3% of the adult catch in trap 1 adjacent to the water butts, it is unlikely that the seasonal abundance patterns of *Cx. pipiens* will be impacted significantly by co-occurrence with such low populations of *C. annulata*.

Adult *Cx. pipiens* were only trapped reliably in one trap (trap 1) next to the water butts (trap 1 averaged 4.96 mosquitoes per night, traps 2, 3 and 4 averaged 0.36 mosquitoes per night), so model simulations are compared to catch data from trap 1. Traps 2, 3 and 4 were further from water butt breeding sites than trap 1, which may account for their lower catch sizes (Fig. 1). A total of 481 mosquitoes were caught in trap 1 over the trapping period, comprising 452 *Cx. pipiens* (94.0%), 26 *C. annulata* (5.4%), 2 *Anopheles maculipennis* (0.4%) and 1 *Aedes geniculatus* (0.2%).

### *Culex pipiens* seasonal abundance patterns

The seasonal abundance pattern for the egg stage shows two clear generational peaks in late April and late June (Fig. 3a). A similar pattern is observed in larvae, with the abundance peaks occurring a few days after the peaks in egg abundance (Fig. 3b). The larval data also shows signs of a small third peak in August. The pupal data clearly shows three peaks in abundance through the year, with a third peak in late August/September that is not obvious in the egg stage (Fig. 3c). The differences in the relative abundances of the larval and pupal populations, particularly in autumn where there is almost no discernible larval peak, indicates that larval survival must be highly variable throughout the season. This strongly suggests that density-dependent population regulation may be driving abundance patterns throughout the year with the cessation of egg-laying in late July and August leading to reduction in larval density and a consequent decrease in larval competition. It is also possible that the reduction in larval abundance at this time leads to a reduction in the predator population and a release in larval mortality due to predation.

The adult data shows less defined generations than the immature data (Fig. 3d) with a small peak early in the season upon emergence from diapause, followed by a drop in numbers (note that despite the missed collections there were still only two recorded mosquitoes on the five nights of trapping from 27th April to 7th May) before increasing again throughout late May and June. The less pronounced initial peak in adults than immature stages is likely to be partly explained by the later start date of adult versus immature sampling as the first eggs were observed 6 days before the first night of adult trapping. High numbers of adults are sustained throughout July before catch sizes decrease again throughout August as they enter diapause. There is no evidence of a third peak in adults, as observed in larvae and pupae. This is likely to be because the adult stage lasts over twice as long as the immature stage on average [5, 91] and this relatively long adult lifespan leads to considerable overlap in cohorts of adults arising from batches of eggs laid by different females, particularly in the middle to the end of the adult season (i.e. 2nd and 3rd generation individuals each year). Each of these adult cohorts develops and survives at different rates depending on the fluctuating environmental conditions to which they have been subjected during their lifetimes, causing a blurring of generational peaks in the adult population. Further, adults emerging from the final pupal peak would be programmed for diapause, meaning they will not host-seek and will not be present in traps [92].

We hypothesise that the gap of almost two months between the early-season and mid-season immature peaks (Fig. 3a-c) is caused by the death of aged females that have oviposited in spring after over-wintering, followed by a slower egg-to-adult development of this first spring generation. The energetic requirements of over-wintering can weaken adult mosquitoes [93], resulting in adult females dying immediately after egg-laying. This is supported by the observation of high numbers of dead adult female *Cx. pipiens* floating on the surface of the water butts in the early part of the season. Predictions of the stage durations from the DDE model, using observed water temperatures, suggest that the time required for development through all life stages in the spring is 40–70 days. This time delay is congruent with the observed duration of approximately 50 days between the first and second egg peaks in the data (Fig. 3a). The time between egg peaks cannot be explained by the time required for adults to locate a blood meal and complete a gonotrophic cycle between egg-laying events, which the DDE model estimates to take less than 20 days at the observed spring air temperatures. This high mortality of post-diapause females was added to the DDE model as an additional seasonal mortality rate and is shown in Additional file 1: Text S2.1.

**Fig. 4:**
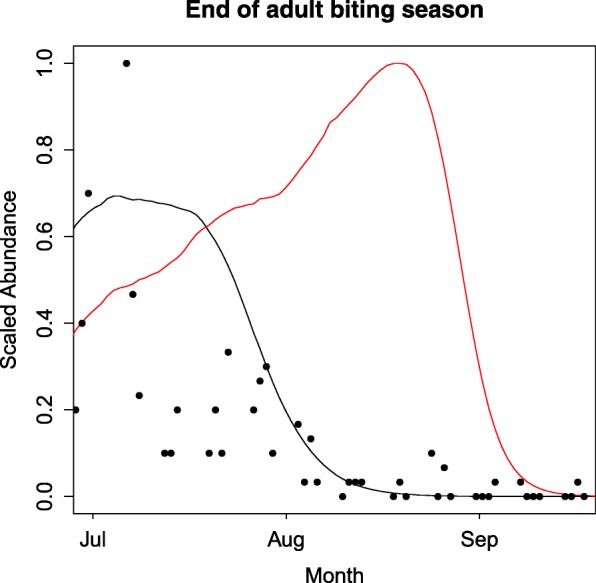
Biting season thresholds. The adult abundance predicted by the DDE model is shown under the Ewing et al. [62] autumn photoperiod threshold (red line) and the updated photoperiod threshold which brings diapause initiation forward by approximately one month (black line, Additional file 1: Table S1), with the field observations as data points

The field data showed that the mosquito biting season, when adults could be caught in host-seeking traps, ended in late July into early August, as seen in Fig. 4 (data points and black line). This contrasts prior estimates of the mosquito biting season by Ewing et al. [62], which were based on monitoring of mosquitoes entering and leaving diapause shelters (Fig. 4, red line). Thresholds based on appearance in diapause shelters resulted in a longer active mosquito season than observed in the field, with mosquito activity predicted to end approximately one month later than observed using host-seeking traps (Fig. 4, red line). This mismatch in timings likely stems from the fact that emergent adults feed on nectar before entering diapause shelters [92, 94] and are therefore not host-seeking or attracted to traps and will not transmit disease. At the start of the season any discrepancy was small; however, diapause exit was brought forward by one week as to match the observed first week of that mosquitoes were observed in traps. Consequently, by confronting the DDE model with the field data we were able to better understand two key elements of the adult dynamics, which were not captured from the literature alone.

### Role of density-independent and density-dependent mortality factors: which factors best predict abundance patterns?

**Fig. 5:**
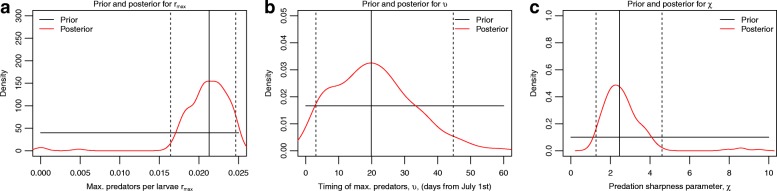
Posterior distributions for predation parameters. Panels **a**, **b** and **c** show the prior (black) and posterior (red) distributions for *r*_*max*_, *υ* and χ respectively. The solid vertical lines show the posterior medians and the dashed vertical lines show the 95% credible intervals. Posterior medians were: *r*_*max*_ = 0.0214, *υ* = 19.84 and *χ* = 2.45

The ABC fitting procedure, with the competition parameters estimated from Madder et al. [2], resulted in posterior distributions for the three seasonal predation parameters shown in Fig. 5. The distributions highlight that there is a relatively tight range of values for *r*_*max*_ and χ which were most likely to lead to the observed field data. The posterior median of *r*_*max*_ corresponds to a predator-prey ratio of 1:47 when the predator population is at its peak. The timing parameter, *υ*, showed a credible interval running from early July to mid-August, although it is most likely that the predator peak occurred in late July. The posterior median of χ means that the sinusoidal wave governing the predator-prey ratio is raised to a power of 2.45. When extending the ABC fitting procedure to include the competition parameters, there was no clear change to the posterior distributions of the predation parameters. The posterior distributions of the competition parameters showed little change when compared to their priors and the median estimates of the competition parameter values were very similar to those fitted to experimental data from Madder et al. [2] (Additional file 1: Figure S4 and Text S6): *c*_0_ (experimental) = 0.00319; *c*_0_ (ABC) = 0.00446; *c*_1_ (experimental) = 0.00469; *c*_1_ (ABC) = 0.00490. The fact that the posterior distributions of the competition parameters showed little evidence of convergence towards a particular value indicates that the choice of competition parameters is less influential on the fit of the model to the data than the choice of predation parameters (which showed greater convergence), within the range of the priors chosen.

**Fig. 6:**
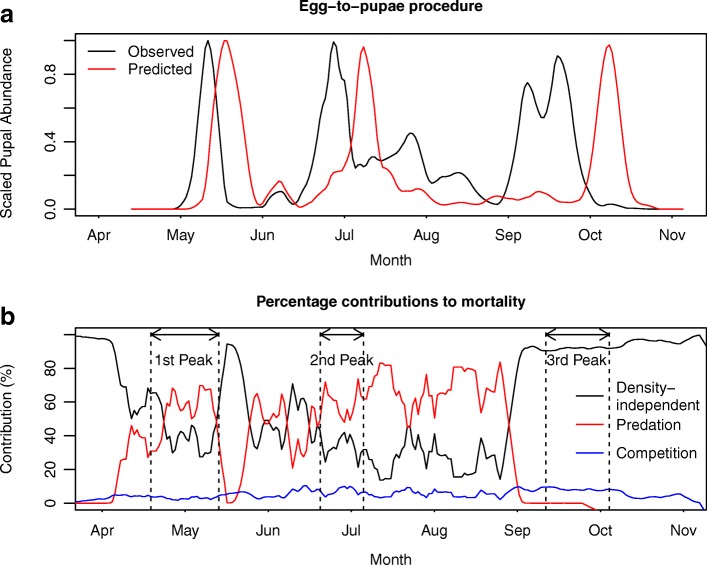
Fit of egg-to-pupae procedure. **a** The fit of the egg-to-pupae procedure predictions to the data, where the black line shows the observed pupal abundance and the red line shows the pupal abundance predicted by the egg-to-pupae model under the posterior median values of the predation parameters. **b** The relative contributions of density-independent mortality (black, Ewing et al. [62] Equation S.6), predation (red, Equation A8) and competition (blue, Equation A10) to the total estimated mortality throughout the season. A centered 7-day moving average of each time series was taken to smooth out some of the substantial daily variability in the data. The windows within the dotted lines show the duration of immature development leading up to the pupal peaks predicted by the model. For each peak the second dotted line corresponds to the time, *t*_*peak*_, of the maximum predicted pupal abundance for that peak and the first dotted line corresponds to the time at which the eggs which gave rise to the peak were predicted to be laid by the model, using the delay given in Equation 1. Parameter values are given in Additional file 1: Table S1.

Using the median posterior values for each of the predation parameters and the competition estimates from Madder et al. [2], Fig. 6a shows that the observed and predicted pupal abundances under the egg-to-pupae procedure show good agreement both in the number of peaks captured and in the relative sizes of those peaks. Figure 6b shows the estimated relative contributions of the different sources of larval mortality throughout the year. The vertical dotted lines show the estimated duration of immature development leading up to the predicted pupal peak, to show what combination of larval mortality sources gave rise to the peak predicted by the model. Observing the fit of the egg-to-pupae procedure (Fig. 6a in conjunction with Fig. 6b) the model proposes that the first two pupal peaks appear to be shaped both by density-independent mortality and by predation, with competition for resources contributing little to the overall mortality. Between these two peaks there is a substantial reduction in estimated density-dependent mortality in mid-to-late May, with density-independent mortality dominating. This corresponds to the end of the initial peak in larvae causing density-independent mortality to dominate. Similarly, the final peak in the autumn is driven by a release in predicted density-dependence, with the predator population crashing and almost all mortality becoming attributable to the temperature-dependent, density-independent mortality. Note that some caution should be used in interpreting these results because as the discrepancy in timing between the observed and predicted pupal peaks increases, the reliability of using the contributions to larval mortality from the model predictions to make inferences about the field data may decrease. However, as the relative sizes of all three pupal peaks are accurately captured, the mortality patterns proposed by the model certainly show a viable process by which the observed pupal peaks could have been generated.

Figure 6a shows that the model is overestimating the duration of immature development, such that the peaks are predicted to occur later in the season than was observed in the field. Further, this discrepancy in timings increases as the season progresses (first peak - 5 days late, second peak - 12 days late, third peak - 19 days late). This increase in stage duration could not be attributed to any steady change across the season in egg, larval or pupal stage durations, as none showed steady increases through the year (Additional file 1: Figure S5 and Text S7). Further, whilst the assumption that the predator-prey ratio followed a sinusoidal pattern is slightly restrictive, a more flexible predation function would not shorten the stage durations, nor would we expect it to shift the predicted pupal peaks earlier in the year such that the agreement between predictions and observations would improve. Consequently, the overestimation of the stage duration is thought to be likely to stem from the fact that larval development rates are known to be affected by both intra- and interspecific competition for resources [91, 95, 96]; however, these effects have not been incorporated into the model and no direct measures of nutritional content were taken at the water butts.

**Fig. 7:**
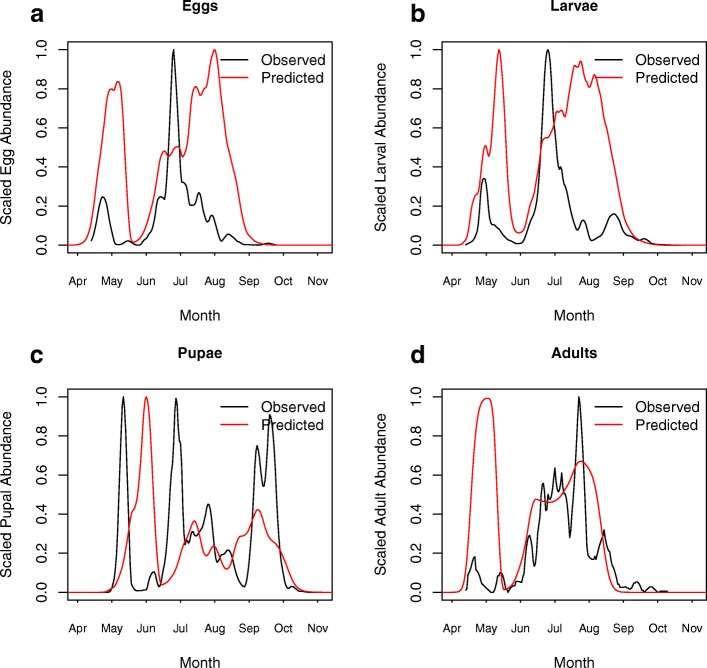
Comparison of DDE Model and Data. The field data (black line) from butt 4 is shown against the full DDE model predictions (red line). Panels **a, b, c** and **d** show the scaled 7-day moving averages of the field and model-predicted abundances for eggs, larvae, pupae and adults, respectively. Parameter values are given in Additional file 1: Table S1

The full DDE model was also run using the posterior median predation values (Fig. 5), with the results shown in Fig. 7. Panels a and b show that the general form of the abundance profile for eggs and larvae is captured by the model, with two main peaks in both field observations and model predictions. However, in both cases the DDE model overestimates the duration and size of the peaks, meaning that the initial peak estimated by the model is predicted to be larger than was observed in the field. This increased width of the immature population peaks is believed to stem from the assumption made that eggs are laid at a constant rate. In reality, eggs are laid by adults in batches separated by the length of the gonotrophic cycle and this pulsed behaviour would likely lead to the stronger generational peaks observed in the field data with less overlap between cohorts. This is supported by the observation in Fig. 6a that the egg-to-pupae procedure did not overestimate the width of the peaks in this way because we extrapolated forward directly from the egg observations made in the field, rather than relying on the egg abundance predicted by the DDE model. The inaccuracies in the egg and larval stages have knock-on effects for the pupal stage, where the model captures the number of peaks but not the relative sizes of those peaks. As was observed with the egg and larval populations, we propose that the shortcomings observed here stem from errors in the model-predicted egg-laying rate and the subsequent egg and larval abundances. These errors are then propagated through to the pupal stage.

Despite some shortcomings in the DDE model estimation of immature abundances, the adult abundance patterns are well captured by the DDE model for the majority of the year, with only the size of the initial peak overestimated by a factor of 5 by the DDE model. The ability of the model to capture the adult dynamics well, despite the model being parameterised from the literature data, much of which is concerned with capturing immature vital rates, lends support for this general modelling approach despite its limitations. It is also noteworthy that, even if seasonality in the ratio of predators to prey is removed, a strongly seasonal abundance pattern remains (Additional file 1: Figure S6 and Text S8) and is driven by a range of factors, such as photoperiod effects on diapause behavior, temperature impacts of development and mortality and age-dependent mortality of post-diapause females.

## Discussion

By collecting seasonal abundance data for all stages of the mosquito life-cycle at a high temporal resolution and integrating this with a mathematical population model, we were able to investigate the relative contributions of density-independent and density-dependent processes to mosquito seasonality at a field site in southern England. In the studied field system, the model predicted that density-independent mortality and interspecific predation were both important determinants of abundance patterns with varying roles throughout the year (Fig. 6b). Conversely, larval competition was not estimated to be particularly influential in shaping patterns of immature survival in the permanent field habitat examined (Fig. 6b), with the choice of competition parameters observed to have less influence on our ability to capture seasonal abundance patterns than the choice of predation parameters (Additional file 1: Figure S4). This is likely to be due to the very high organic matter content in the water butts stemming from the hay infusion (Fig. 6a) [2, 11, 34]. Combining the field data with the DDE model also allowed us to better understand patterns of adult mortality, which was observed to be very high after egg-laying of the first spring generation. Further, when the model was tuned with diapause timings from Ewing et al. [62] we observed that timings deduced from *Cx. pipiens* appearance in overwintering shelters [6] led to an overestimation of the mosquito biting season (Fig. 4) because mosquitoes feed on nectar between adult emergence of the last generation and appearance in shelters [92]. Whilst patterns of immature survival and development were captured quite well, as shown by the egg-to-pupae procedure, we observed that the fit of the full DDE model to the data was quite poor in parts of the season across the immature stages. However, despite inaccuracies in the DDE model estimation of the patterns of immature abundance, the pattern of adult abundance was captured well after overestimating the size of the initial peak (Fig. 7). This highlights the importance of validating mosquito population models used to assess control strategies on data from relevant life stages. Using standard approaches of comparing only adult abundance predictions to adult data [48, 51, 52] does not guarantee that larval abundances are correctly predicted by a given model. Policy makers evaluating larval control strategies may therefore have unjustified confidence in recommended control programs as the discrepancy between immature predictions and observation cannot necessarily be inferred from adult data alone.

Integrating the DDE model with data from all mosquito life stages allowed more informative conclusions to be drawn regarding the precise processes that were captured well by the model versus those which were not. The egg-to-pupae procedure shown in Fig. 6a suggests that the model accurately captures the patterns of immature survival, as the relative sizes of all three pupal peaks are accurately reproduced. Were the model to be validated against only adult data, it would not be possible to carry out the egg-to-pupae procedure to confirm if these processes are parameterised correctly and to explore the relative contributions of density-dependence and density-independence throughout the season. The egg-to-pupae procedure, and indeed the immature field data alone, aid our ability to identify particular features of the mosquito phenology and to attribute those to biological phenomena. Whilst validation against adult field data can be valuable, particularly since it is adults which transmit disease, it is important to consider that adult data are likely to be noisier than immature data due to the wide range of factors influencing adult capture probability. Specifically, weather conditions will affect adult flight activity and therefore capture probability on a given night [97], adults will only appear in traps when they are host-seeking [63] and not during other phases of the gonotrophic cycle, trap height has been shown to affect catch sizes of *Cx. pipiens* [63], and adult *Cx. pipiens* will disperse at distances of up to 3 km [98] such that sample populations are not closed. Conversely, immature mosquitoes are confined to a particular site and the capture probability of each life stage is likely to remain relatively constant across the year. This allows seasonal abundance patterns to be observed more directly in immature life stages than in adult life stages, as observed in the field data (Fig. 3).

Using the egg-to-pupae procedure we estimated that mortality due to intraspecific competition was relatively unimportant when compared to density-independent mortality and mortality from predation at our field site (Fig. 6b) and that the choice of competition parameters had less impact on our ability to reproduce abundance patterns (Additional file 1: Figure S4). It is probable that the proposed relative lack of importance of competition in this case stems from the fact that we monitored large, permanent habitats that were observed to be colonised by predators early in the season. Given the size of the habitats, which were infused with hay for months prior to spring oviposition, it is likely that resource availability was high, which would explain the observed low mortality levels due to competition. Further, once predators have colonised a site, those predators may regulate abundance, such that larval competition will have little impact on survival. We suggest that competition is likely to be more influential in small, transient, predator-free larval development sites, which may appear after periods of heavy rainfall. This idea is supported by the findings of Washburn et al. [99] who find that mosquito larvae in permanent ground pools are typically limited by natural enemies, whereas those in transient containers tend to be limited by resource availability. Were exact predator numbers to have been recorded and organic matter content to have been quantified then it would certainly have been more straightforward to determine the dominant drivers of immature mortality, however this was not possible within the mesocosms used in the field set up, which were designed to monitor natural fluctuations in *Cx. pipiens* seasonal abundance as closely as possible.

Whilst competition for resources was not predicted to have a profound impact on larval survival at the field site, it is probable that it affects the larval stage duration. Intra- and interspecific competition for food and co-occurrence with predators have all been shown to increase the larval development time in a range of mosquito species, including *Cx. pipiens* [34, 76, 80, 96]. However, the development rate relationships in the model depend only on temperature due to the difficulty in quantifying the effect of food availability and/or predator presence on development rates. Figure 6a shows that the model is overestimating the duration of immature development, as the peaks in predicted pupal abundance from the DDE model occur after those observed in the field. This mismatch in timings is relatively small in the early part of the season (5 days difference between the spring peaks) and steadily increases through the year (approximately 19 days difference between the autumn peaks). The mismatch could not be explained by stage duration predictions from the DDE model, which did not show an increasing or decreasing trend through the year (Additional file 1: Figure S5). The inclusion of variable food availability, or predator co-occurrence, on larval stage durations in dynamic population models remains a relatively unexplored area due to the practical difficulties of measuring and quantifying this effect in field situations but one which may prove fruitful in understanding this mismatch in development times. One model that includes effects of variable nutrition levels, and that future models could built upon is Skeeter Buster, which is a spatially explicit modelling tool for studying *Ae. aegypti* populations [100] and which incorporates temporally and spatially variable food availability. However, in addition to modelling food depletion by consumption, Skeeter Buster requires the external food input, a daily decay factor and the conversion of dead immatures to nutritional resources to be defined for each larval habitat within a given study area, which will both increase complexity and reduce generality of the model.

The effect of temperature on larval mortality and development rates of various mosquito species, including *Cx. pipiens* have been well studied in laboratory settings [2, 3, 5, 34, 101]. We have shown that when a DDE model was parameterised only from these existing laboratory studies, it was able to accurately capture patterns of immature survival (Fig. 6a). Further, the estimates of the larval competition parameters from fitting the model to field data showed good agreement with the estimates from experimental work (Additional file 1: Figure S4). The main areas in which the model fails to capture dynamics are: immature stage duration, overwintering and adult egg-laying. The steadily increasing mismatch in immature stage durations has been discussed in detail in the previous paragraph. The majority of adults which enter diapause do so without taking a blood meal, having emerged from pupae in the autumn programmed for diapause [92]. Conversely, older, parous individuals rarely overwinter [92]. This process is not captured in the DDE model, which assumes an equal temperature-dependent mortality rate for all adults regardless of age. This appears to cause an overestimation of the size of the diapausing population and a consequent overestimation of the size of the spring population (Fig. 7d). If the adult stage were split into sub-stages this would allow a separate diapausing class to be included with a separate mortality, thus adjusting for the reduced number of adults which overwinter.

The DDE model also predicts the summer peak in the egg and larval populations to be longer than was observed in the field data (Fig. 7a, b). Egg-laying and larval abundance in the field data show strong generational peaks and a decreased egg population in mid-to-late July and early August (Fig. 7a, b), whilst the model predicts high egg and larval abundance throughout all of July. This discrepancy between the observed and predicted egg and larval numbers stems from the fact that the model assumes egg-laying is spread out over the length of the gonotrophic cycle, whilst in reality eggs should be laid in pulses at the end of each gonotrophic cycle [68, 102]. This pulsed egg-laying behaviour could be achieved by splitting the adult stage into multiple sub-stages to reflect whether they are egg-laying or undergoing a gonotrophic cycle, as in [46–48]. However, splitting the adult stage to account for various gonotrophic cycles becomes problematic when the model is further extended to explicitly incorporate disease transmission, due to the requirement to track both the gonotrophic cycle and the extrinsic incubation period (EIP) simultaneously. Tracking both processes at once within this modelling framework would require the relationship between biting rates and progression of the EIP under variable temperatures to be well understood. Such work has been carried out for some mosquito-borne diseases such as malaria and dengue [103, 104]; however, these relationships are not well studied for WNV.

We have shown that the DDE model, which was constructed almost entirely from data available in the literature, overestimates the relative size of the adult population upon diapause emergence but captures the observed adult seasonality in the middle and end of the active season well. This highlights the need for an improved understanding of how to quantify over-wintering mortality and diapause fitness costs, which will constrain vector seasonality and are vital for understanding disease persistence between years [105]. There are some notable shortcomings in the estimation of immature abundances, which emphasize the importance of validating models across all life stages of the species of interest if we are to understand the role of abiotic versus biotic processes in driving population dynamics and develop models that can make reliable inferences about the impacts of control measures targeted at a particular life stage. The DDE model shows that the inclusion of density-dependent mortality in the larval stage is crucial in both regulating population size and driving abundance patterns. Density-dependence has also been shown to have profound impacts on mosquito control measures as ill-timed interventions can lead to releases in density-dependence and consequent increases in mosquito abundance [41]. Consequently, greater attention should be given to modelling of density-dependent larval processes, which are largely neglected in vector and disease models at present [45]. By incorporating the range of density-dependent and density-independent processes in models and by appropriately validating these models using data across the life stages of the species of interest we can improve confidence in the application of these models to assess vector control or predict disease dynamics.

The seasonal abundance data were collected over one season, which is a shorter time than would be desired to provide robust evidence confirming the presence, absence or relative importance of particular ecological processes. However, the substantial challenges and resources required to collect datasets of sufficient duration to prove ecological processes in the field are substantial, as is clear from the lack of availability of high resolution multi-stage datasets currently available for vector species. Existing long-term studies only monitor single life stages at lower temporal resolutions, which means that complex seasonal dynamics can be missed and the underlying causes of these seasonal patterns are poorly understood [106, 107]. Consequently, we feel that collecting and analysing a dataset with such high temporal resolution across the life stages has provided valuable insights into the mosquito ecology and will provide useful guidelines and information to help shape further, more long-term studies. Further studies of this nature are needed to confirm the findings presented in this study and to provide a more robust understanding of how patterns of density-dependent and density-independent processes interact to shape seasonal abundance. In this study, parameters estimating the seasonal variation in the ratio of predators to prey were estimated based on a single year of data from a single location, contributing to uncertainty in the model. With a more extensive dataset over multiple years and locations it would be possible to assess how the importance of these density-dependent and density-independent processes, both during the active season and over the diapause season, may vary temporally and geographically over the *Cx. pipiens* range and in different landscape contexts, with knock-on consequences for mosquito-borne disease transmission and persistence between years [108].

## Conclusions

By challenging a novel mathematical model with high temporal resolution field data monitoring all life stages of *Cx. pipiens* our model predicted that density-independent mortality and interspecific predation on larvae interacted to shape patterns of mosquito seasonal abundance in a permanent aquatic habitat. We propose that competition for resources is likely to become a more influential driver of mosquito survival where periods of high rainfall create transient habitats. Combining a rich field dataset with a vector population model also improved our understanding of the initiation and termination of the mosquito biting season, which will have implications for the timing and length of potential disease transmission seasons. Finally, we highlight that challenging vector population models with data from all life stages is important if reliable inferences are to be made, especially in the context of modelling mosquito control measures, which often target individual life stages.

## Additional files

Additional file 1: **Text S1**-**S8.** A summary of the DDE model framework, followed by a detailed discussion of where the model departs from Ewing et al. [62]. Information regarding parameter values and the method for solving the DDEs are also given, along with details of the dynamic time warping applied to compare model predictions with field data, details of the ABC fitting not shown in the main text, model output of immature stage duration and a description of predicted dynamics under the constant predation scenario. **Figure S1.** Predator seasonal forcing: the seasonal forcing function, *r*(*t*), is shown, highlighting how changes to ν and χ affect the ratio of predators to larvae throughout the season. **Figure S2.** Larval Competition: the exponential function fit to the Madder et al. [3] data is shown (*R*^2^ = 0.96). **Table S1.** Parameter values used to run the DDE model simulations. **Figure S3.** Mappings applied by the DTW mapping algorithm. a The indices of the field observations on the x-axis and the model predictions on the y-axis; the red line shows the relationship if no time warping were applied. b The points on each time series mapped onto their corresponding points on the other. **Figure S4.** Prior and posteriors from ABC fitting: the priors and posterior distributions for the ABC fitting run with all three predation parameters, *r*_*max*_, ν and χ, and both competition parameters c_0_ and c_1_. **Figure S5.** Immature stage durations based on the hourly temperature data from butt 4. **Figure S6.** Model results under constant predation: the field data (black line) from butt 4 is shown against the full DDE model predictions (red line) with the ratio of predators to prey held constant by setting χ = 0. The scaled abundances presented are 7-day moving averages of the field and model-predicted abundances. (PDF 299 kb)

Additional file 2: Counts of each life stage, along with data on larval identification and the temperatures in the water butts. (XLSX 270 kb)

Additional file 3: **Text S9.** A full description of the data collection procedure is given along with a detailed description of the data presented in Additional file 2. (PDF 709 kb)

## Abbreviations

ABC: Approximate Bayesian computation
CEH: Centre for Ecology & Hydrology
DDE: Delay differential equation
DTW: Dynamic time warping
WNV: West Nile virus
