# Supplementary material for: Uncovering mechanisms behind mosquito seasonality by integrating mathematical models and daily empirical population data: Culex pipiens in the UK
Source: Parasit Vectors. 2019 Feb 7;12:74. doi: 10.1186/s13071-019-3321-2 (PMC6367758; doi:10.1186/s13071-019-3321-2)
Supplement: Supplementary file 1 — Text S1-S8. A summary of the DDE model framework, followed by a detailed discussion of where the model departs from Ewing et al. [62]. Information regarding parameter values and the method for solving the DDEs are also given, along with details of the dynamic time warping applied to compare model predictions with field data, details of the ABC fitting not shown in the main text, model output of immature stage duration and a description of predicted dynamics under the constant predation scenario. Figure S1. Predator seasonal forcing: the seasonal forcing function, r(t), is shown, highlighting how changes to ν and χ affect the ratio of predators to larvae throughout the season. Figure S2. Larval Competition: the exponential function fit to the Madder et al. [3] data is shown (R2 = 0.96). Table S1. Parameter values used to run the DDE model simulations. Figure S3. Mappings applied by the DTW mapping algorithm. a The indices of the field observations on the x-axis and the model predictions on the y-axis; the red line shows the relationship if no time warping were applied. b The points on each time series mapped onto their corresponding points on the other. Figure S4. Prior and posteriors from ABC fitting: the priors and posterior distributions for the ABC fitting run with all three predation parameters, rmax, ν and χ, and both competition parameters c0 and c1. Figure S5. Immature stage durations based on the hourly temperature data from butt 4. Figure S6. Model results under constant predation: the field data (black line) from butt 4 is shown against the full DDE model predictions (red line) with the ratio of predators to prey held constant by setting χ = 0. The scaled abundances presented are 7-day moving averages of the field and model-predicted abundances. (PDF 299 kb) [file 13071_2019_3321_MOESM1_ESM.pdf]

# Additional File 1 - Uncovering mechanisms behind mosquito seasonality by integrating mathematical models and daily empirical population data: *Culex pipiens* in the UK

## S1 DDE Model Framework

This section gives a summary of the DDE model framework. Further details of the model framework are given by Ewing et al. [1]. The four state equations which correspond to eggs, $E(t)$, larvae, $L(t)$, pupae, $P(t)$ and adults $A(t)$ at time $t$, are

$$\begin{aligned}
\frac{dE}{dt} &= R_E(t) - M_E(t) - \delta_E(T(t))E(t), \\
\frac{dL}{dt} &= R_L(t) - M_L(t) - (\delta_L(T(t)) + \delta_{DD}(L(t)))L(t), \\
\frac{dP}{dt} &= R_P(t) - M_P(t) - \delta_P(T(t))P(t), \\
\frac{dA}{dt} &= R_A(t) - \delta_A(T(t))A(t),
\end{aligned} \tag{S1}$$

where $T(t)$ gives the temperature at time $t$, $\delta_i(T)$ ($i = E, L, P, A$) represents the stage-specific, density-independent, temperature-driven, mortality rate, and $R_i(t)$ and $M_i(t)$ represent the rate of recruitment to and maturation from stage $i$ respectively. The density-dependent mortality term, $\delta_{DD}(L(t))$, incorporates mortality through predation, $\delta_\pi(L(t))$, as in the Ewing et al. [1] model, but also allows for the addition of larval competition, $\delta_{LC}(L(t))$, and can be written as $\delta_{DD}(L(t)) = \delta_\pi(L(t)) + \delta_{LC}(L(t))$. The maturation equations are defined as

$$\begin{aligned}
R_E(t) &= b(t,T)A(t), \\
M_E(t) = R_L(t) &= R_E(t - \tau_E(t))S_E(t)\frac{g_E(T(t))}{g_E(T(t - \tau_E(t)))}, \\
M_L(t) = R_P(t) &= R_L(t - \tau_L(t))S_L(t)\frac{g_L(T(t))}{g_L(T(t - \tau_L(t)))}, \\
M_P(t) = R_A(t) &= R_P(t - \tau_P(t))S_P(t)\frac{g_P(T(t))}{g_P(T(t - \tau_P(t)))},
\end{aligned} \tag{S2}$$

with $g_i(T(t))$ as the development rate at temperature $T(t)$, $b(t,T)$ as the egg-laying rate, $\tau_i(t)$ and $S_i(t)$ as the survival of individuals in stage $i$ ($i = E, L, P$) at time $t$ respectively. The proportion of individuals which survive from recruitment into one class, to maturation to the next, is defined by the following sequence of DDEs,

$$\frac{dS_E}{dt} = S_E(t)\left(\frac{g_E(T(t))\delta_E(T(t-\tau_E(t)))}{g_E(T(t-\tau_E(t)))} - \delta_E(T(t))\right),$$
$$\frac{dS_L}{dt} = S_L(t)\left[\left(\delta_{DD}(t-\tau_L(t)) + \delta_L(T(t-\tau_L(t)))\right)\left(\frac{g_L(T(t))}{g_L(T(t-\tau_L(t)))}\right) - \delta_{DD}(L(t)) - \delta_L(T(t))\right],$$
$$\frac{dS_P}{dt} = S_P(t)\left(\frac{g_P(T(t))\delta_P(T(t-\tau_P(t)))}{g_P(T(t-\tau_P(t)))} - \delta_P(T(t))\right). \tag{S3}$$

Lastly, the rate of change of the duration of each life stage is given by

$$\frac{d\tau_i(t)}{dt} = 1 - \frac{g_i(T(t))}{g_i(T(t-\tau_i(t)))}, \tag{S4}$$

and the duration of the gonotrophic cycle, $\tau_G(t)$, is given by

$$\frac{d\tau_G(t)}{dt} = 1 - \frac{g_G(T(t))}{g_G(T(t-\tau_G(t)))}. \tag{S5}$$

# S2 New and updated functional forms

## S2.1 Adult mortality rate

Motivated by the field observations, we hypothesise that old, post-diapause females experience an increased mortality due to the negative costs which diapause can exert on fitness [2]. The functional form for the adult death rate, $\delta_A(t)$, has been modified from Ewing et al. [1] to incorporate an additional post-diapause death term, which is supported by the decreased adult abundances observed in May, following the initial peak in abundances upon diapause emergence. This term was added to the Gaussian function used in Ewing et al. [1]. The updated adult death rate function is given by

$$\delta_A(t, T(t)) = \begin{cases} \overbrace{\alpha_A T(t)^{\eta_A}}^{\text{old}} + \overbrace{\left(\frac{\Gamma}{\sqrt{2\pi\sigma^2}} \exp\left(-\frac{(t-\tau_G(t)-\mathcal{D})^2}{2\sigma^2}\right)\right)}^{\text{new process}}, & T(t) > \left(\frac{b_{da}}{\alpha_A}\right)^{\frac{1}{\eta_A}} \\ \underbrace{b_{da}}_{\text{updated parameter}}, & \text{otherwise} \end{cases}. \tag{S6}$$

Here $\alpha_A$ and $\eta_A$ are constants fitted to data from the literature regarding the temperature dependence of adult longevity, as in Ewing et al. [1]. $\Gamma$ is a scaling parameter defining the strength of the post-diapause mortality effect, $\sigma^2$ controls the length of time over which this post-diapause mortality acts and $\mathcal{D}$ is the day of the year on which an arbitrary threshold value (80%) of adults have exited diapause. The death rate was constrained not to drop below a base death rate of $b_{da}$, which determines the mortality of diapausing females. The value for $\Gamma$ was chosen such that the post-diapause death rate was sufficient to wipe out the adult population surviving from the previous year. The mortality duration parameter, $\sigma^2$, was chosen to maximise the correlation between the adult field data and the model-predicted adult abundance over the duration which the adult mortality acted (in late May) by increasing $\sigma^2$ in increments of 0.1 and choosing the best fit. The diapause exit parameter, $\mathcal{D}$, was chosen to coincide with the end of the first adult abundance peak in the field data, when it was assumed that the majority of the population had left diapause.

### S2.2 Gonotrophic cycle

A logistic functional form was chosen for the gonotrophic cycle development rate because this gave a better fit to the data [3–5] than the logarithmic functional form originally presented by Ewing et al. [1] (adjusted increased from $R^2 = 0.89$ to $R^2 = 0.90$), whilst including fewer parameters. The logistic functional form is also differentiable at all points, where the original function was not. The logistic functional form is given by,

$$g_G(T(t)) = q_1/(1 + q_2 \exp(-q_3 T(t))), \tag{S7}$$

where $g_G(T(t))$ is the rate of progression of the gonotrophic cycle at temperature $T(t)$, with $q_1, q_2$ and $q_3$ as fitted constants.

### S2.3 Larval competition

Mortality due to intra-specific larval competition has been shown to occur in a range of mosquito species [3, 6–8]. Linear, exponential, quadratic and log-linear functional forms were considered for the mortality rate due to larval competition, with the exponential and quadratic forms being shown to give the best fit to *Cx. pipiens* data presented by Madder et al. [3] (Residual standard errors (RSE) for each form were used as the $R^2$ statistic is not valid for the nonlinear exponential model: exponential RSE= $0.0077$, quadratic RSE= $0.0077$, linear RSE= $0.015$, log-linear RSE= $0.020$). The exponential form was chosen over the quadratic to prevent negative mortality rates at low larval densities. Consequently, larval competition is represented by

$$\delta_{LC}(L(t)) = c_0 \exp\left(c_1 \frac{L(t)}{V}\right), \tag{S8}$$

where $c_0$ and $c_1$ are constants fitted by nonlinear least squares fitting. The exponential function fit to the Madder et al. [3] data is shown in Figure S1. Note that the underlying temperature dependent mortality rate was accounted for before fitting the death rate due to competition.

### S2.4 Seasonally forced predation

The strength of seasonal predation is affected by the ratio of predators to prey, the attack rate of predators and the predators' handling time. The three parameters have high uncertainty, as attack rates and handling times will vary greatly between predator species [9] and the ratio of predators to prey will vary seasonally, by location, and by species [10]. Similar to mosquito development and survival, predator attack rates and handling times may also be affected by temperature. As used by Ewing et al. [1], the larval death rate due to predation is given by the Holling type II function [11, 12]

$$\delta_\pi(L(t)) = \frac{a\mathcal{P}(t)}{V + ahL(t)}. \tag{S9}$$

where $a$ is the attack rate, $h$ is the handling time, $V$ is the volume of habitat and $\mathcal{P}(t)$ is the predator density at time $t$. Under a "constant predation" scenario it is assumed that $\mathcal{P}(t) = rL(t)$, such that the predator density is equal to a constant proportion of the larval density. In considering seasonally forced predation, we make the extension that the proportion of predators to larvae, $r$, varies seasonally. Consequently, $r$ is replaced by $r(t)$ and predator density is related to larval density, according to

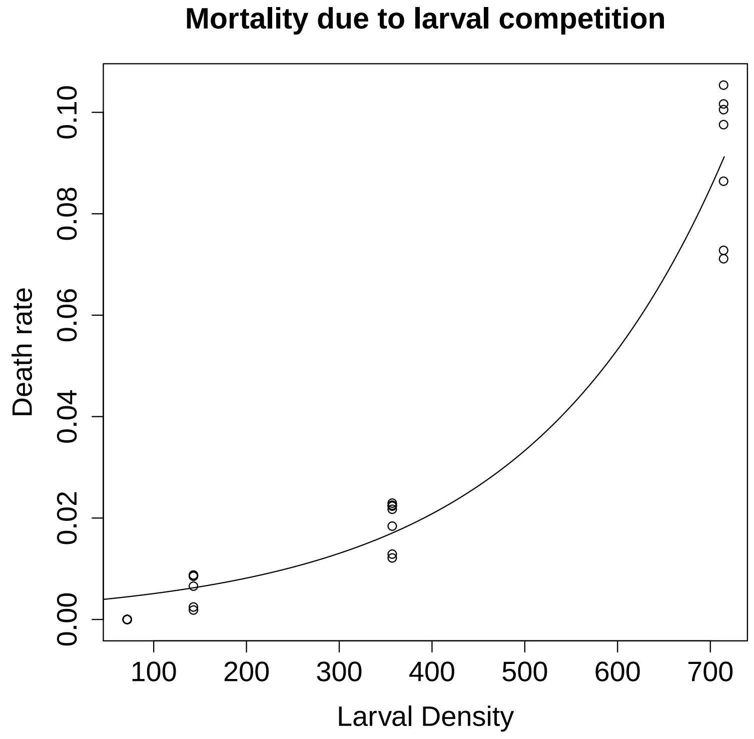


**Figure S1: Larval Competition**: The exponential function fit to the Madder et al. [3] data is shown ($R^2$ = 0.96).

$$\mathcal{P}(t) = r(t)L(t) = r_{max}\left(\frac{1}{2} + \frac{1}{2}\sin\frac{2\pi(t-\upsilon)}{365}\right)^{\chi} L(t), \tag{S10}$$

where $r(t)$ is the number of predators per larva at time $t$, $r_{max}$ is the maximum number of predators per larva, $\upsilon$ defines the time at which the predation peak occurs and $\chi$ defines the time period over which predation is high, as displayed in Figure S2. We assume a fixed volume of larval habitat as the volume of habitat in the field setup was fixed throughout the study. The attack rate and handling time of predators was chosen using studies on common UK predators of *Cx. pipiens* [13], whilst $r_{max}$, $\upsilon$ and $\chi$ were fitted to the field data using ABC rejection sampling.

# S3 Parameter values

The full set of parameter values used in the simulations is given in Table S1.

# S4 Initial history, innoculation and solver code

As in Ewing et al. [1], we solve the system of DDEs in Fortran 90 using the DDE solver (DDE_SOLVER) written by Thompson et al. [20]. The code for the model described here can be found at Ewing et al. [21]. The historical values for the system are as described in Ewing et al. [1], with all stages assumed to be empty for $t < 0$ and all temperatures assumed to be constant at $5°C$. To initiate the system we assume that some innoculation takes place at t = 0. This consists of adding $I_0$ individuals into the adult class ($I_0$ = 5000 in this case). Simulations were begun on the 1st of January and run for 24 months, with the last 12 months of simulated values used for comparison with the field data.

| Parameter | Definition | Value | Reference |
|---|---|---|---|
| $\alpha_E$ | Fit parameter in egg maturation | $2.20 \times 10^{-3}$ | Ewing et al. [1] |
| $\beta_E$ | Fit parameter in egg maturation | 1.77 | Ewing et al. [1] |
| $\alpha_L$ | Fit parameter in larval maturation | $3.15 \times 10^{-3}$ | Ewing et al. [1] |
| $\beta_L$ | Fit parameter in larval maturation | 1.12 | Ewing et al. [1] |
| $\alpha_P$ | Fit parameter in pupal maturation | $7.11 \times 10^{-4}$ | Ewing et al. [1] |
| $\beta_P$ | Fit parameter in pupal maturation | 1.89 | Ewing et al. [1] |
| $\mu_{0E}, \mu_{0L}, \mu_{0P}$ | Baseline immature death rate | 0.0157 | Ewing et al. [1] |
| $\mu_{1E}, \mu_{1E}, \mu_{1P}$ | Optimum temperature for immature survival | 20.5 | Ewing et al. [1] |
| $\mu_{2E}, \mu_{2E}, \mu_{2P}$ | Width parameter for immature death rate | 7 | Estimated from laboratory data |
| $\alpha_A$ | Fit parameter in adult death | $2.17 \times 10^{-8}$ | Ciota et al. [14] |
| $\beta_A$ | Fit parameter in adult death | 4.48 | Ciota et al. [14] |
| $b_m$ | Baseline maturation rate | $\frac{1}{60}$ | Almirón et al. [15] and Loetti et al. [16] |
| $b_{di}$ | Threshold immature death rate | 1 | Time-scale of model |
| $b_{da}$ | Baseline adult death rate | 0.003 | Sulaiman et al. [17], Onyeka et al. [18], and Bailey et al. [19] |
| $c_0$ | Fit parameter in competition | 0.00319 | [3] |
| $c_1$ | Fit parameter in competition | 0.00469 | [3] |
| $a$ | Attack rate of predators | 1.03 | Onyeka [13] |
| $h$ | Handling time of predators | 0.043 | Onyeka [13] |
| $V$ | Volume of larval habitat | 20l | By calculation |
| $R$ | Egg raft size | 200 | Vinogradova [4] |
| $q_1$ | Gonotrophic cycle fit parameter | 0.202 | Ewing et al. [1] |
| $q_2$ | Gonotrophic cycle fit parameter | 74.5 | Ewing et al. [1] |
| $q_3$ | Gonotrophic cycle fit parameter | 0.246 | Ewing et al. [1] |
| $\xi_S$ | Spring diapause threshold | 13.7 | From fieldwork |
| $\xi_A$ | Autumn diapause threshold | 15 | From fieldwork |
| $\omega_S$ | Spring diapause transition | 5 | From fieldwork |
| $\omega_A$ | Autumn diapause transition | 3.5 | From fieldwork |
| $\Gamma$ | Post-diapause mortality mult. | 8 | From simulation |
| $\sigma^2$ | Post-diapause mortality duration | 4 | From simulation |
| $\mathcal{D}$ | 80% diapause exit threshold day of year | 109 | From fieldwork |
| $r_{max}$ | Peak predators per larva | 0.0214 | From ABC fitting |
| $\upsilon$ | Predation timing parameter | 19.84 | From ABC fitting |
| $\chi$ | Predation sharpness parameter | 2.45 | From ABC fitting |
| $\varphi$ | Latitude | 51.6 | Latitude of Wallingford field site |

**Table S1:** Parameter values used to run the DDE model simulations.

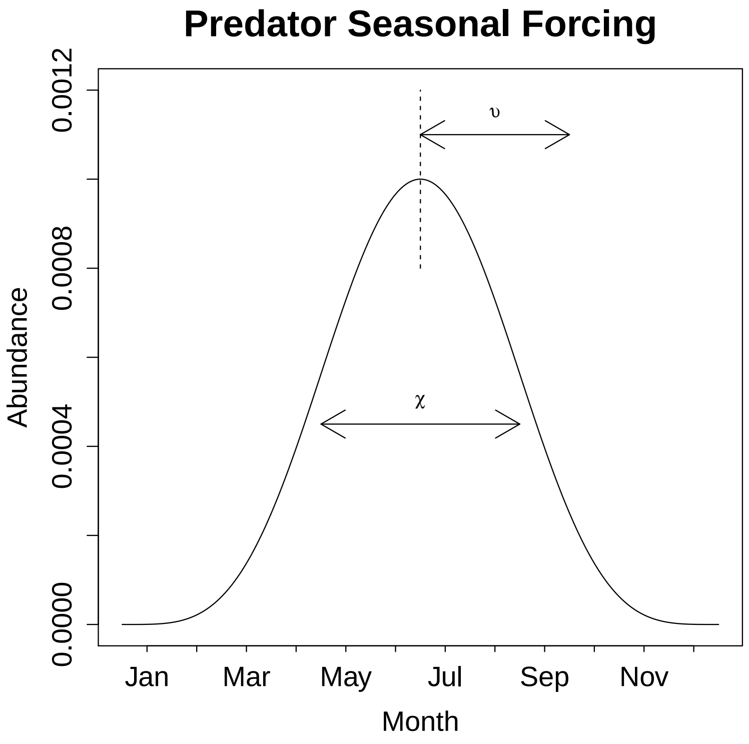


**Figure S2: Predator seasonal forcing**: The seasonal forcing function, $r(t)$, is shown, highlighting how changes to $\upsilon$ and $\chi$ affect the ratio of predators to larvae throughout the season.

# S5 Dynamic time warping

Dynamic time warping [22] was used to compare the predicted and observed patterns of abundance as the timing of features in the data did not always align in time. Consequently, traditional measures like route mean squared error (RMSE) were not able to assess the ability of the model to capture features in the data when the stage duration was either over- or under-estimated. The warping applied in comparing the fitted egg-to-pupae procedure to the field data is shown in Figure S3.

# S6 ABC fitting including competition

The priors and posterior distributions for the three predation parameters and both competition parameters are shown in Figure S4. In all cases the difference between the parameter estimates from fitting only predation parameters using ABC and fitting predation and competition parameters using ABC is small.

# S7 Immature stage durations

The immature stage durations at all time points throughout the season are shown in Figure S5. Note that none of the immature stage durations are seen to steadily increase throughout the season, which suggests that the steadily increasing discrepancy between observed and predicted stage durations in Figure 6 (a) does not stem from one specific stage duration being increasingly overestimated through the year.

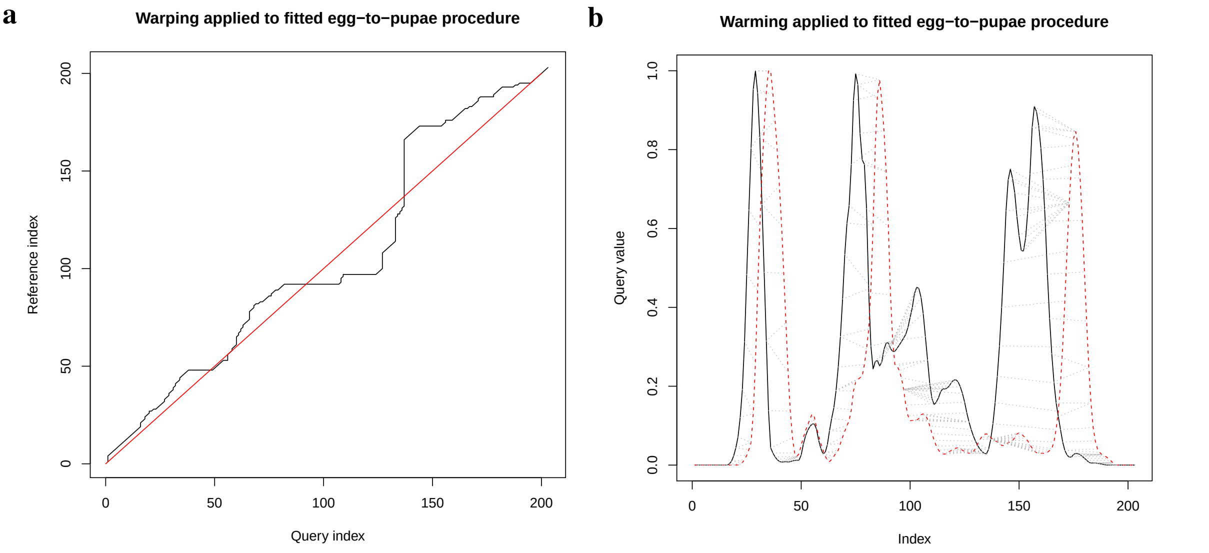


**Figure S3: DTW mapping:** The mappings applied by the DTW mapping algorithm are shown. Plot (a) shows the indices of the field observations on the $x$-axis and the model predictions on the $y$-axis and the red line shows the relationship if no time warping were applied. Plot (b) shows the points on each time series mapped onto their corresponding points on the other.

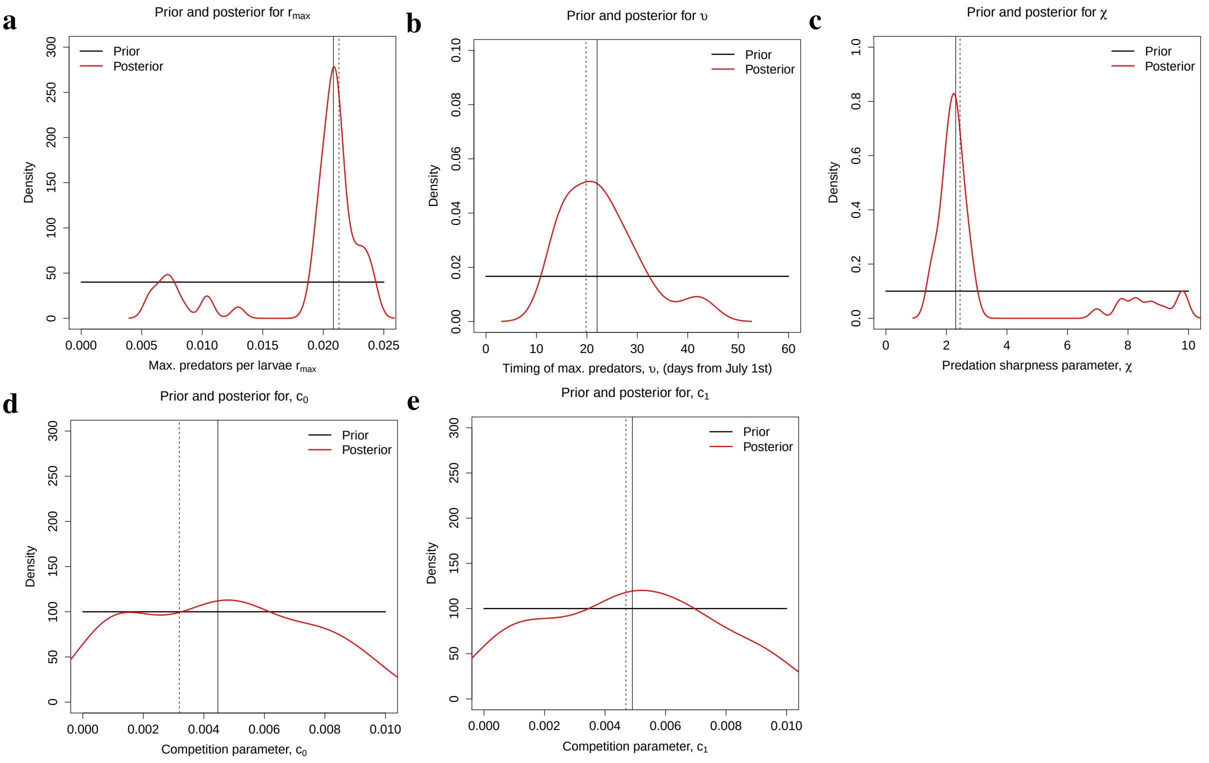


**Figure S4: Prior and posteriors from ABC fitting:** The priors and posterior distributions for the ABC fitting run with all three predation parameters, $r_{max}$, $\upsilon$ and $\chi$, and both competition parameters $c_0$ and $c_1$ are shown. The solid vertical lines show the median of the posterior distribution in each case. In the case of the predation parameters, the dotted vertical lines show the median of the posterior distribution fitted using the experimentally derived competition parameters. The vertical dotted lines on the plots for the competition parameters show the experimentally derived estimates.

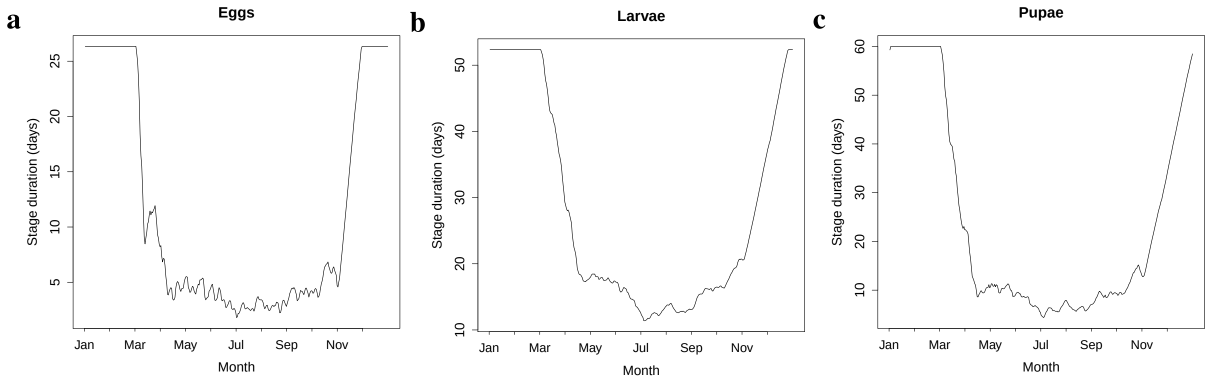


**Figure S5: Immature stage durations:** The immature stage durations based on the hourly temperature data from butt 4 are shown.

# S8 Seasonality under constant predator to prey ratio

The extent to which the addition of a seasonally forced predator-prey ratio was influencing the observed pattern of seasonal abundance was investigated by comparing the results of the DDE model (Figure 7) with the output when the ratio of predators to prey was held constant by setting $\chi = 0$.

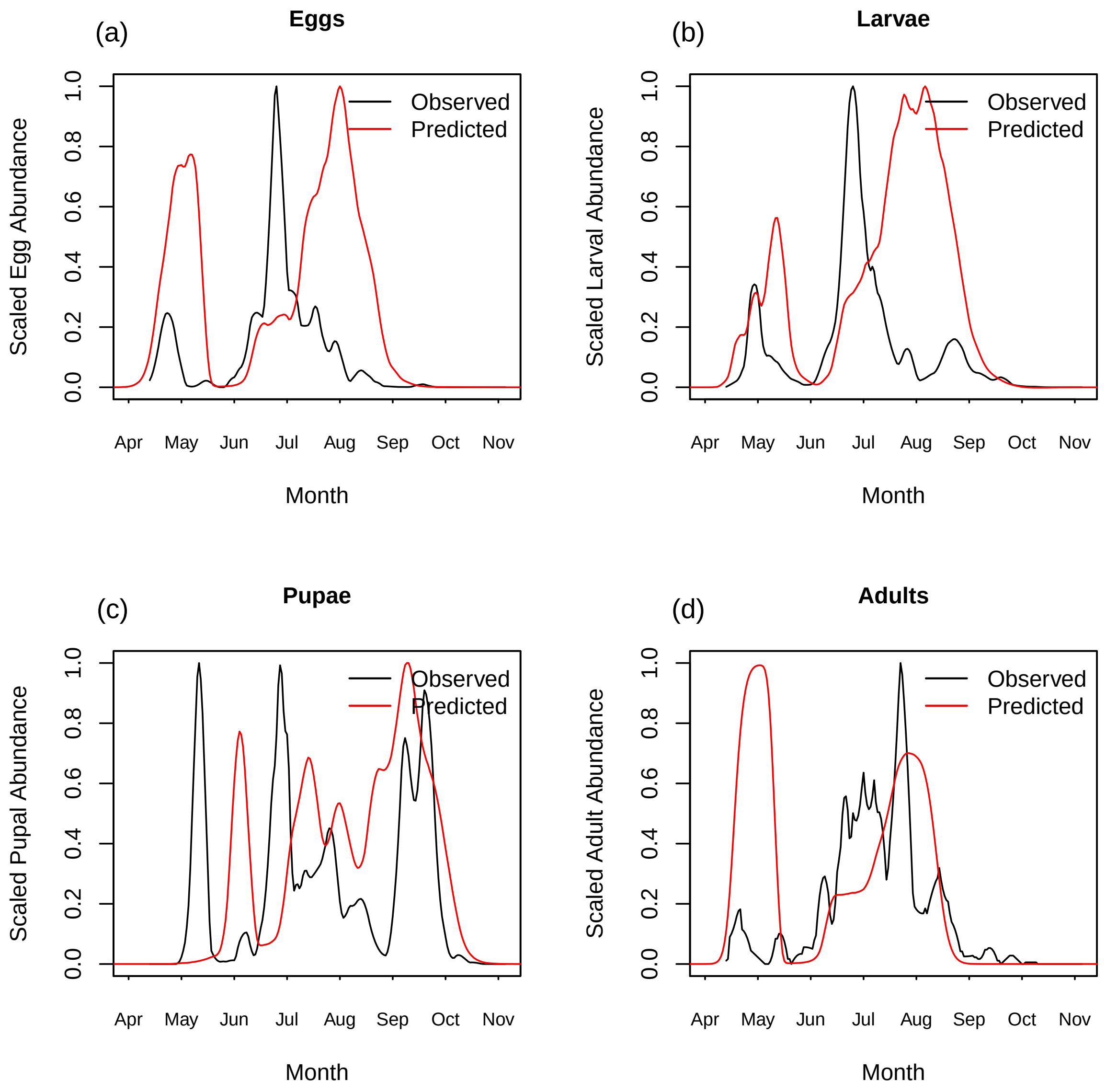


**Figure S6: Model results under constant predation:** The field data (black line) from butt 4 is shown against the full DDE model predictions (red line) with the ratio of predators to prey held constant by setting $\chi = 0$. The scaled abundances presented are 7-day moving averages of the field and model-predicted abundances.

## References


[1] D. A. Ewing et al. “Modelling the effect of temperature on the seasonal population dynamics of temperate mosquitoes”. In: *Journal of Theoretical Biology* 400 (2016), pp. 65–79. ISSN: 10958541. DOI: 10.1016/j.jtbi.2016.04.008. URL: http://dx.doi.org/10.1016/j.jtbi.2016.04.008.

[2] D. A. Hahn and D. L. Denlinger. “Meeting the energetic demands of insect diapause: nutrient storage and utilization”. In: *Journal of Insect Physiology* 53.8 (Aug. 2007), pp. 760–73. ISSN: 0022-1910. DOI: 10.1016/j.jinsphys.2007.03.018.

[3] D. J. Madder, G. A. Surgeoner, and B. V. Helson. “Number of generations, egg production, and developmental time of *Culex pipiens* and *Culex restuans* (Diptera: Culicidae) in Southern Ontario”. In: *Journal of Medical Entomology* 20.3 (1983), pp. 275–287.

[4] E. B. Vinogradova. *Culex pipiens pipiens mosquitoes: taxonomy, distribution, ecology, physiology, genetics, applied importance and control*. 2nd ed. PENSOFT, 2000.

[5] D. M. Hartley et al. “Effects of temperature on emergence and seasonality of West Nile virus in California.” In: *The American Journal of Tropical Medicine and Hygiene* 86.5 (May 2012), pp. 884–94. ISSN: 1476-1645. DOI: 10.4269/ajtmh.2012.11-0342.

[6] P. Agnew, C. Haussy, and Y. Michalakis. “Effects of density and larval competition on selected life history traits of *Culex pipiens quinquefasciatus* (Diptera : Culicidae)”. In: *Journal of Medical Entomology* 37.5 (2000), pp. 732–735.

[7] S. M. Muriu et al. “Larval density dependence in *Anopheles gambiae s.s.*, the major African vector of malaria.” In: *The Journal of Animal Ecology* 82.1 (Jan. 2013), pp. 166–74. ISSN: 1365-2656. DOI: 10.1111/1365-2656.12002.

[8] J. Couret, E. Dotson, and M. Q. Benedict. “Temperature, larval diet, and density effects on development rate and survival of *Aedes aegypti* (Diptera: Culicidae).” In: *PloS ONE* 9.2 (2014), e87468. ISSN: 1932-6203. DOI: 10.1371/journal.pone.0087468.

[9] H. Quiroz-martinez and A. Rodriguez-Castro. “Aquatic insects as predators of mosquito larvae”. In: *The American Mosquito Control Association* 23.2 (2007), pp. 110–117.

[10] J. M. Medlock and K. Snow. “Natural predators and parasites of British mosquitoes–a review”. In: *European Mosquito Bulletin* 25.April (2008), pp. 1–11. URL: http://e-m-b.org/sites/e-m-b.org/files/EMB25_1.pdf.

[11] G. A. Marti et al. “Predation efficiency of indigenous larvivorous fish species on *Culex pipiens* L. larvae (Diptera: Culicidae) in drainage ditches in Argentina.” In: *Journal of Vector Ecology* 31.1 (2006), pp. 102–6. ISSN: 1081-1710.

[12] S. Fischer et al. “Effect of habitat complexity on the predation of *Buenoa fuscipennis* (Heteroptera: Notonectidae) on mosquito immature stages and alternative prey”. In: *Journal of Vector Ecology* 38.2 (2013), pp. 215–223.

[13] J. O. A. Onyeka. “Studies on the natural predators of *Culex pipiens* L. and *C. torrentium* Martini (Diptera: Culicidae) in England”. In: *Bulletin of Entomological Research* 73.02 (1983), pp. 185–194. ISSN: 0007-4853. DOI: 10.1017/S0007485300008798.

[14] A. T. Ciota et al. “The effect of temperature on life history traits of *Culex* mosquitoes”. In: *Journal of Medical Entomology* 51.1 (2014), pp. 55–62.

[15] W. R. Almirón and M. E. Brewer. “Winter biology of *Culex pipiens quinquefasciatus* Say , (Diptera : Culicidae) from Córdoba , Argentina”. In: *Memórias do Instituto Oswaldo Cruz* 91.5 (1996), pp. 649–654.

[16] V. Loetti, N. Schweigmann, and N. Burroni. “Development rates, larval survivorship and wing length of *Culex pipiens* (Diptera: Culicidae) at constant temperatures”. In: *Journal of Natural History* 45.35-36 (Sept. 2011), pp. 2207–2217. ISSN: 0022-2933. DOI: 10.1080/00222933.2011.590946.

[17] S. Sulaiman and M. W. Service. “Studies on hibernating populations of the mosquito *Culex pipiens* L. in southern and northern England”. In: *Journal of Natural History* 17.6 (1983), pp. 849–857. ISSN: 0022-2933. DOI: 10.1080/00222938300770661.

[18] J. O. A. Onyeka and P. F. L. Boreham. “Population studies, physiological state and mortality factors of overwintering adult populations of females of *Culex pipiens* L. (Diptera: Culicidae)”. In: *Bulletin of Entomological Research* 77 (1987), pp. 99–111.

[19] C. L. Bailey et al. “Winter survival of blood-fed and nonblood-fed *Culex pipiens L.*” In: *The American Journal of Tropical Medicine and Hygiene* 31.5 (1982), pp. 1054–61. ISSN: 0002-9637.

[20] S. Thompson and L. Shampine. “A Friendly Fortran DDE Solver”. In: *The Third International Conference on the Numerical Solution of Volterra and Delay Equations*. 2004, pp. 1–23.

[21] D. A. Ewing et al. *Temperate-Mosquito-DDE v1.0*. 2016. DOI: 10.5281/zenodo.48525. URL: http://zenodo.org/record/48525.

[22] M. Muller. “Dynamic Time Warping”. In: *Information Retrieval for Music and Motion*. 2007.
