# Supplementary material for: Uncovering mechanisms behind mosquito seasonality by integrating mathematical models and daily empirical population data: Culex pipiens in the UK
Source: Parasit Vectors. 2019 Feb 7;12:74. doi: 10.1186/s13071-019-3321-2 (PMC6367758; doi:10.1186/s13071-019-3321-2)
Supplement: Supplementary file 3 — Text S9. A full description of the data collection procedure is given along with a detailed description of the data presented in Additional file 2. (PDF 709 kb) [file 13071_2019_3321_MOESM3_ESM.pdf]

Additional File 2: Text S9 - Culex pipiens seasonal abundance at Wallingford field site

Overview

This dataset contains details of the seasonal abundance of each of the life stages of the mosquito species, *Culex pipiens,* at a field site located at the Centre for Ecology & Hydrology in Wallingford. Immature life stages (eggs, larvae and pupae) were monitored three times per week from March until October 2015, whilst adult counts were taken four times per week from April until October in the same year.

Experimental design/sampling regime and collection methods

The field study was carried out on the grounds at the Centre for Ecology & Hydrology (CEH) site in Wallingford (51˚ 36' 9.0144" N, 1˚ 6' 45.7344" W).

Immature *Cx. pipiens* were monitored using four 450 litre circular water butts placed at locations in close proximity at the Wallingford field site (Figure 1). Butts number 1 and 2 were placed in exposed locations with some cover from bushes on the north side but all other sides open and no overhanging vegetation. These butts received direct sunlight throughout the majority of the day. Butt number 3 was more sheltered with no overhanging vegetation but cover on the north and west sides and direct sunlight until late afternoon or early evening dependent on the time of year. Butt number 4 was sheltered under a tree and only received direct sunlight in the morning. All butts were located within 20 yards of each other. In January the butts were filled with clean water, which was then infused with hay by suspending 2kg of hay in a net bag in each butt. The bags of hay were left in the butts until the end of March and then remove. A HOBO temperature logger, floating on the surface of the water, was set to record surface water temperature at hourly intervals in each butt. The number of egg rafts in each butt was counted at 10am on Mondays, Wednesdays and Fridays. Samples were taken from the 2nd of March until the 5th of October 2015. On each sampling occasion, after the egg rafts were counted a 500ml dip was taken from each of the north, south, east and west edges of each water butt using the standard dipping procedure (described in Additional File 2 of (Fillinger et al., 2008)). No dips were taken from the middle of the butt as both larvae and pupae were observed to congregate at the edges. The dips were transferred into one or more white plastic trays for counting. When numbers were less than approximately 20, numbers of 1st/2nd instar larvae, 3rd/4th instar larvae, and pupae were counted in the field at the time of collection. In most cases this was not possible due to the large number of individuals in each sample, so photographs were taken of the contents of each tray. The samples were then counted manually on the computer using Microsoft Paint. All samples were returned to the water butts after photographing to prevent removal effects from one catch to the next. Twenty 4th instar larvae were taken from each water butt (when

abundances were high enough that the number removed was a small proportion of the total population) monthly for morphological identification to species level. Those 4th instar larvae taken for identification were examined by microscopy in the laboratory (Becker et al., 2003). Larvae were killed prior to examination by submersion in boiling water.

To sample the adult population four John W. Hock Miniature Downdraft Blacklight (UV) traps (also referred to as CDC light traps) were run nightly from the 14th of April until the 2nd of October, when there had been 5 consecutive empty collections, in the yellow locations spread around the Wallingford field site (Figure 1). The traps were baited with dry ice to attract female adult mosquitoes and were run 4 times a week overnight from Monday to Thursday throughout the year (though seven nights were missed due to logistical issues). Trap 1 was hung amongst some trees adjacent to the water butts and traps 2 to 4 were hung in the tree line at the side of an 200 adjacent field used to graze cattle at distances of approximately 80m, 140m and 200m from the water butts. The traps were run from 1700 each day until 0900 the following morning. Adults were placed in the freezer immediately after collection and left for at least one hour before identification. All mosquitoes caught were identified to species level by microscopy in the laboratory (Becker et al., 2003) and the number of females of each species was recorded. Males were not recorded.

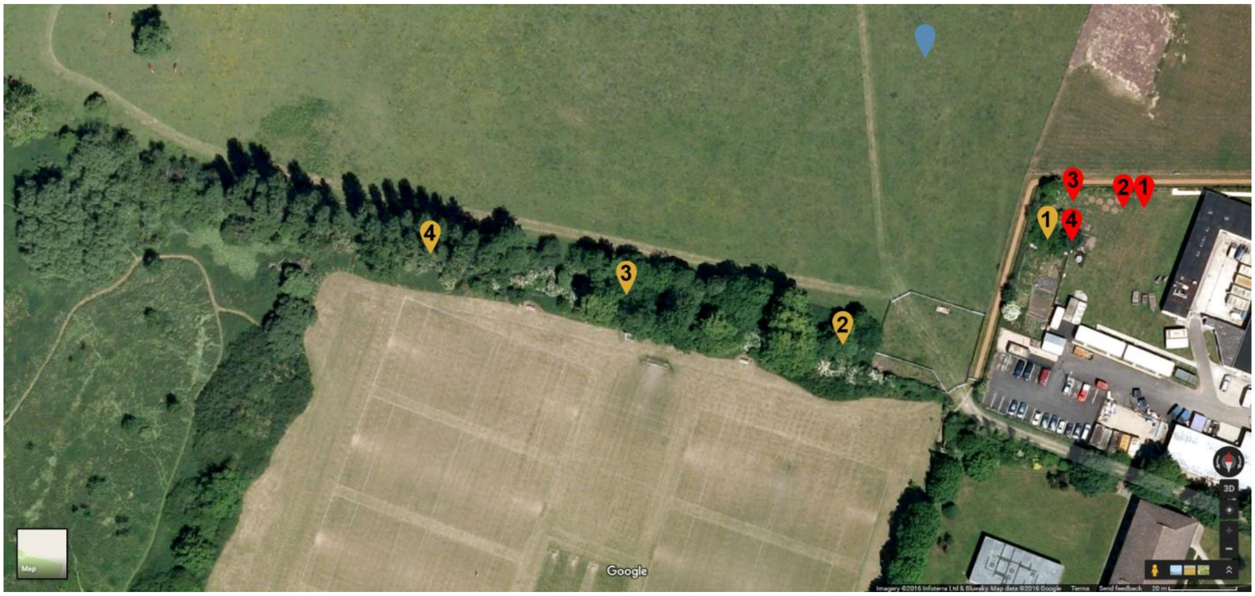

Figure 1: CEH Wallingford field site. The red markers show the locations of water butts 1-4 for immature sampling. The yellow markers show the locations of adult traps 1-4. The blue marker shows the location of the adjacent CEH weather station.

Fieldwork and laboratory instrumentation

For adult sampling John W. Hock Miniature Downdraft Blacklight (UV) traps baited with dry ice were used. Identification was carried out in the laboratory using an assortment of standard dissection and light microscopes.

Nature and units of recorded values

All egg abundance data is presented as counts for egg rafts, split by water butt. Larval and pupal data is given as the number of larvae or pupae counted per sampling occasion, split by water butt. Adult data gives the number of adult female *Cx. pipiens* accounting on each sampling occasion, split by trap. Temperature data is given in degrees Celsius.

Quality control

Larval and pupal numbers were calculated by taking photos of the contents of each dip and counting on Microsoft paint. The validity of counting from photos was checked by comparing direct counts from the tray with counts from photos on the first two days of sampling and the estimates were observed to be consistent.

Details of data structure

This dataset comprises five sheets entitled – “Eggs”, “Larvae and Pupae”, “Adults”, “Larval Identification” and “Water Temperatures”.

Eggs

Each row contains details of the number of egg rafts counted on a particular day.

Column contents:

Date: gives the date on which the count was taken
Butt n: denotes which butt (n=1,2,3,4) the count was taken from.

Larvae and Pupae

Each row contains the details of the number of larvae and pupae counted on a particular day. The columns are partitioned such that B-P gives Butt 1 data, Q-AE gives Butt 2 data, AF-AT gives Butt 3 data and AU-BI gives Butt 4 data. Columns are arranged to denote whether columns relate to larval or pupal counts, with appropriate headings.

Column contents:

Date : gives the date on which the count was taken
N,E,S,W: denotes whether which “corner” of the butt the sample was taken from
L1/2: denotes a count for $1^{st}/2^{nd}$ instar larvae
L3/4: denotes a count for $3^{rd}/4^{th}$ instar larvae

Adults

Each row contains the details of the number of adult female mosquitoes caught by a particular trap on a particular date. Entries with “NA” indicate occasions where the trap could not be run due to a staff shortage or dry ice delivery issues.

Column contents:

Date : gives the date on which the count was taken
Location: denotes which trap the count corresponds to (for comparison with Figure 1, WB – 1, F1 – 2, F2 – 3, F3 – 4).
Cx. pipiens: gives the number of adult female culex pipiens identified
Cs. annulata: gives the number of adult female culiseta annulata identified
Ae. Geniculatus: gives the number of aedes geniculatus identified

Larval Identification

This file gives details of the dates at which identification of 4th instar larvae was carried out, the number of larvae identified on each occasion, how many of these larvae were identified as *Cx. pipiens* and which butts the identified individuals came from.

Water Temperatures

This file gives hourly water temperature measurements from the surface of each water butt from the 3rd of March until the 2nd of November 2015.

Column contents:

Date: gives the date on which the measurement was taken
Time: gives the time at which the measurement was taken
Butt: gives the water butt from which the measurement was taken (in degrees Celsius).
